# Propionic Acid Remodels Mitochondrial Metabolism in SH-SY5Y Cells

**DOI:** 10.3390/biology15161424

**Published:** 2026-08-18

**Authors:** Caitlyn Mahony, Erin Buchanan, Colleen O’Ryan

**Affiliations:** 1 Department of Molecular and Cell Biology, University of Cape Town, Cape Town 7700, South Africa; c.mahony@uct.ac.za (C.M.); bcheri005@myuct.ac.za (E.B.); 2 Neuroscience Institute, University of Cape Town, Cape Town 7700, South Africa

**Keywords:** mitochondrial dysfunction, propionic acidemia, autism spectrum disorder, inherited metabolic diseases, respirometry, confocal microscopy

## Abstract

Mitochondrial mechanisms are increasingly implicated in complex neurological conditions, including Autism Spectrum Disorder (ASD). Propionic acid (PPA) is widely used to study mitochondrial dysfunction in preclinical models of ASD. However, the molecular mechanisms that drive PPA-induced neurotoxicity are unresolved. Here, we examined mitochondrial remodeling under PPA-induced stress in neuroblastoma SH-SY5Y cells. PPA systemically altered the transcriptional regulation of mitochondrial dynamics and disrupted canonical proteins involved in mitochondrial fusion (L-OPA1, MFN2), fission (DRP1) and quality control (LC3-II). Confocal microscopy revealed an upregulation of both fission and fusion events and impairments to mitochondrial integrity, connectivity and turnover. Live-cell respirometry demonstrated consequent deficits in both oxidative and glycolytic energy production, while respiratory chain electron flow assays illustrated a shift in TCA cycle flux driven by a remodeling of mitochondrial substrate utilization. This work describes a molecular signature of metabolic stress in the SH-SY5Y system, providing novel insights into the mechanisms and manifestations of PPA-induced neurotoxicity.

## Simple Summary

This study seeks to better understand how neurological disorders are linked to mitochondrial dysfunction. We examined how mitochondria respond to metabolic stress in neuroblastoma cells by looking at changes to important genes and proteins required for mitochondrial health. We found disturbances to several proteins that maintain the shape and flexibility of mitochondrial networks. This was supported by increased network remodeling, leading to mitochondrial fragmentation and degradation. At the same time, stressed mitochondria changed how they used different metabolic pathways and produced significantly less energy. Together, this work helps us to understand how neurons respond to metabolic stress through mitochondrial remodeling. Since mitochondrial network morphology and metabolism are critical for neurodevelopment and function, understanding these mechanisms could help us to treat brain disorders caused by mitochondrial dysfunction.

## 1. Introduction

Mitochondria are emerging as important players in molecular neuroscience, with critical roles in neurodevelopment, physiology and function [1]. Increasing evidence implicates a mitochondrial component in the etiology of neurodegenerative [2], neuropsychiatric [3] and neurodevelopmental [4] conditions, including autism spectrum disorder (ASD) [5]. Concurrently, inherited metabolic diseases (IMDs) frequently manifest with neurological phenotypes associated with neurodevelopmental disorders [6]. This mechanistic convergence raises the intriguing potential of repurposing mitochondrial biomarkers and therapies to diagnose and mitigate neuropathology [7]. However, the mechanisms of how mitochondria contribute to ASD etiology remain poorly understood. Thus, diverse preclinical models have been established to explore the mechanistic relationship between mitochondrial dysfunction and neurodevelopment. Propionic acid (PPA) is a short-chain fatty acid that is widely used to mimic ASD-like behaviours in vivo [8]. The PPA murine model is associated with impaired cognition, memory, sociability and motor function as well as increased hyperactivity, stereotypy, perseveration, anxiety and seizures [8,9,10]. Interestingly, the accumulation of PPA observed in Propionic Acidemia (PA) is associated with neurological symptoms that are also widely observed in ASD—including cognitive deficits, seizures, dystonia, developmental delay and language impediments [11,12,13]. There is a documented clinical relationship between PA and ASD, while both PA and ASD manifest with immunological and gastrointestinal abnormalities, oxidative stress and mitochondrial dysfunction [5,14]. Preclinical studies have found that PPA inhibits TCA cycle flux, increases oxidative stress [14,15,16] and disrupts neuronal morphology, organization and signaling [17,18,19,20]. However, the mechanisms that drive PPA-induced neurotoxicity are yet to be fully elucidated.

Molecular neuroscience research is inherently constrained by the inaccessibility of brain tissue, yet the functional consequences of molecular disruptions are highly cell-type and life-stage specific. In this context, in vitro models have emerged as useful reductionist systems for exploring specific aspects of PPA-induced neurotoxicity, including work in the SH-SY5Y cell line [21,22,23,24,25,26]. Derived from human neuroblastoma tissue, the SH-SY5Y system is widely used as a cost-effective “workhorse” cell line to study neurodevelopment and disease [27]. Previous studies have established that PPA exerts concentration-dependent effects on the metabolism and viability of undifferentiated SH-SY5Y cells, showing neuroprotective effects at low doses (0.1–10 µM) [21,23] while inducing mitochondrial dysfunction-driven apoptosis from 1–10 mM [22,24,25]. We previously reported significant changes to mitochondrial ultrastructure and dynamics in undifferentiated SH-SY5Y cells exposed to PPA stress [26], adding to cumulative data showing disruptions to mitochondrial biogenesis, morphology and function in this system [22,24,25]. However, the relationship between molecular and metabolic remodeling under PPA stress remains uncharacterized in this context.

The dynamic nature of mitochondrial homeostasis (mitostasis) presents two important considerations for any in vitro study of metabolism. Mitochondria are constantly adapting to intracellular demands, balancing biogenesis, fission, fusion and mitophagy to maintain a dynamic state of homeostasis [28]. Yet, most experimental techniques rely on intrinsically reductionist endpoint measurements of mitochondrial function that seldom reflect the multifaceted nature of upstream processes. As such, a multipronged approach using several complementary techniques is recommended for the study of mitostasis in vitro [29]. Moreover, mitochondrial metabolism is shaped by substrate availability as much as it is impacted by metabolic stress. The composition of cell culture medium should therefore be informed by the context-specific relevance of metabolic state for the central research question at hand.

In this study, we harness the flexible, efficient and cost-effective nature of undifferentiated SH-SY5Y cells to conduct a multifaceted assessment of mitochondrial dynamics. The existing literature on PPA stress in the SH-SY5Y system employs the traditional high-glucose culture medium with serum supplementation recommended for cell-line expansion. Given the well-documented glycolytic nature of the SH-SY5Y cell line, we employed glucose and serum depletion, which has previously been shown to promote an oxidative metabolic profile more reminiscent of neuronal-like cells [30,31]. We examine interdependent disruptions to transcriptional, morphological and metabolic facets of mitostasis under PPA stress that substantially alter endpoint metabolic state. We describe time- and dose-dependent changes to the transcriptional regulation of mitochondrial dynamics in response to PPA, coupled with a remodeling of mitochondrial network integrity, connectivity and turnover. Lastly, we demonstrate the functional consequences to bioenergetic state, highlighting distinct changes to substrate utilization profiles that inhibit both glycolytic and oxidative energy production. Together, these data contribute to a mechanistic understanding of mitochondrial mechanisms in neuropathology that could be harnessed to improve long-term clinical outcomes in ASD and other neurological disorders.

## 2. Materials and Methods

### 2.1. SH-SY5Y Cell Culture

SH-SY5Y cells (ECACC, 94030304) were obtained from Sigma-Aldrich (Pty) Johannesburg, South Africa (Catalogue Number 94030304-1VL) and maintained in a 1:1 ratio of DMEM (Thermo Fisher Scientific LTC, Johannesburg, South Africa, 41965039) and Hams F12 (Thermo Fisher Scientific, 11765054) with 1% Penicillin–Streptomycin (Thermo Fisher Scientific, 15140122), and 15% Fetal Bovine Serum (FBS) (Thermo Fisher Scientific, 10493106) at 5% CO_2_ and 37 °C. Cells were sub-cultured in 25 cm^2^ flasks at 85–95% confluency at a 1:5 ratio using 0.05% Trypsin-EDTA (Sigma-Aldrich, T3924-100ML) followed by a three-minute centrifugation at 1000 rpm (Hemle, Wehingen, Germany, Z446 high capacity centrifuge). All cells used for experiments (passage 19–22) were adapted to Eagle’s Minimum Essential Media (EMEM) (Thermo Fisher Scientific, BoC 1-31S01-I): F12 at a 1:1 ratio to Ham’s F12 with 1% PS, 15% FBS, 1% non-essential amino acids (NEAA) (Inqaba Biotechnical Industries, Tshwane, South Africa, SC 0823) and 2 mM L-glutamine (Inqaba Biotechnical Industries, BoC 5-10K00-H), hereafter referred to as complete EMEM:F12, at least one passage before seeding. Both untreated controls and experimental samples were exposed to a 24 h FBS depletion before treatment. Propionic acid (PPA) was administered in the form of sodium propionate (C_3_H_5_NaO_2_, CAS # 137-40-6) (Sigma-Aldrich, P5436-100G) by dissolving 0.096 g in 10 mL pre-warmed sterile MilliQ water (Merck, Darmstadt, Germany, Millipore Milli-Q water system P15052) to form a 1 M stock solution which was filtered through a 0.22 mm filter and diluted to 3, 5 or 10 mM in serum-free EMEM:F12.

### 2.2. Assessment of Cell Viability

Cell viability was assessed using the Thiazolyl Blue Tetrazolium Bromide (MTT) (Sigma-Aldrich, M5655-500MG) assay according to the protocols recommended by the manufacturer. Briefly, cells were seeded in clear 96-well plates (Sigma-Aldrich, M0812-100EA) at 5 × 10^5^/mL in a final volume of 200 μL per well in complete EMEM:F12, left to adhere overnight and subjected to 24 h of FBS depletion before treatment. After treatment, cells were incubated with 0.5 mg/mL MTT for two hours at 37 °C before the MTT solution was removed and cells were lysed with 100 μL of dimethyl sulfoxide (DMSO) per well. Finally, absorbance was measured at 570 nm using an absorbance spectrophotometer microplate reader (Thermo Fisher Scientific, Multiskan^TM^ GO model 1510). Each treatment was administered in triplicate, and each well was corrected against the average absorbance of DMSO-blanks. Relative cell viability was calculated as the average blank-corrected absorbance of all three triplicates normalized to the average of untreated controls.

### 2.3. RNA and DNA Extraction

For DNA and RNA extractions, SH-SY5Y cells were seeded in 6-well plates at 3 × 10^5^ per well in complete EMEM:F12, allowed to adhere for 24 h and subjected to 24 h of FBS depletion before treatment. After PPA exposure, RNA was extracted using the Quick-RNA^TM^ Miniprep (Zymo Research, Tustin, CA, USA, R1055). Cells were lysed with 300 μL lysis buffer per well and scraped for 1 min before RNA was extracted in accordance with the manufacturer’s instructions, eluted in 25 μL DNAse/RNAse-free water and stored at −80 °C. DNA extractions were carried out according to the protocol described by Aljanabi & Martinez, (1997) [32]. Briefly, cells were incubated in 300 μL lysis buffer (Saline/EDTA; 10 mM EDTA, 40 mM NaCl) for 10 min at 37 °C before being lysed with 10% SDS and 20 mg/mL Proteinase K at 56 °C for 2 h, incubated with 6 M NaCl at room temperature for one hour, centrifuged twice at 10,000 rpm (Eppendorf South Africa, Johannesburg, South Africa, 5415R refrigerated centrifuge, serial number 542601273) for 10 min and incubated with 1 mL isopropanol overnight at −20 °C. Lastly, samples were centrifuged at 10,000 rpm (Eppendorf South Africa, 5415R refrigerated centrifuge) for 20 min before the pellet was washed with 70% ethanol, air-dried for 1 h, eluted in 20 μL TE buffer (10 mM Tris; 1 mM EDT8) and stored at −20 °C. All samples were quantified using a NanoDrop ND-1000 (NanoDrop Technologies LLC, Wilmington, DE, USA) before downstream analysis.

### 2.4. Quantitative Reverse Transcription PCR (RT-qPCR)

All RNA samples used for RT-qPCR analysis had a concentration of ≥95 ng/μL and A260:280/260:230 ratios between 1.8–2.2. The cDNA synthesis was performed using the Tetro^TM^ cDNA Synthesis Kit (Bioline Meridian Sciences, London, UK, BIO-65043) according to the manufacturer’s instructions. A total of 700 ng of RNA was added to each reaction, along with 0.5 μL of oligoDT and 0.5 μL Random Hexamer primers in a total of 20 μL/reaction. cDNA was stored at −80 °C for no longer than 7 days before use. All DNA samples with A260:280 and 260:230 ratios between 1.8–2.2 and 1.5–2.1 respectively were diluted in MilliQ water to 45 ng/μL before downstream qPCR assays. RT-qPCR assays were run using a Rotor-Gene Q6-plex (QIAGEN South Africa, Johannesburg, South Africa) in 10 μL reactions comprising of 5 μL Luna Universal qPCR Master Mix (New England Biosciences, Ipswich, MA, USA, M3003L) and gene-specific quantities of cDNA/DNA and primers detailed in Supplementary File S1, Table S1 along with the relevant cycling conditions. Primers synthesized by Inqaba Biotechnical Industries. Samples from between three and five biological repeats as well as standard curves were run in triplicate, and data was analyzed using Rotor-Gene Q Software 2.3.5. All qPCR runs had efficiencies between 0.8 and 1.2 and R values between 0.90 and 1.2 respectively and the standard deviation between technical replicates was <0.3. Relative expression was calculated relative to the housekeeping gene beta-2-microtubulin (β2M) using the delta delta Ct method where fold change in expression = 2^−ΔΔCt^ [33].

### 2.5. Western Blot Analyses

SH-SY5Y cells were seeded at 2 × 10^5^ cells in T25 flasks, allowed to adhere for 24 h and subjected to a 24 h FBS depletion before treatment with 3, 5 or 10 mM PPA for 24 or 72 h. Cells were lysed with 150 uL extraction buffer containing RIPA buffer (Thermo Fisher Scientific, 89900), 1× Halt Phosphatase Inhibitor (Thermo Fisher Scientific, 78420) and 1× Halt Protease Inhibitor (Thermo Fisher Scientific, 87786), stored at 4 °C for 90 min and centrifuged at 13,000 rpm for 10 min. Supernatants were collected and quantified using the BCA Assay (Thermo Fisher Scientific, 23227) according to the protocol recommended by the manufacturers. Protein samples were diluted 1:1 in a Laemmli buffer containing 95% 2× Laemmli Buffer Stock (Biorad BBRD1610737) and 5% β-mercaptoethanol (Thermo Fisher Scientific, 21985023) before storing at −80 °C until analysis. Protein samples (20 ug per lane) were separated using 12% stain-free protein gels (kit Biorad, 1610185; ammonium persulfate, Sigma-Aldrich, A3678; TEMED, Sigma-Aldrich, T9281) with a 10–180 kDa protein ladder (Thermo Fisher Scientific, 26616) in 1× Tris-Glycine running buffer at 100 V for 10 min followed by 120 V for 60 min. Proteins were transferred to a 0.2 μm PVDF membrane (Sigma-Aldrich, GE10600021) by semi-dry transfer at 15 V for 80 min. PVDF membrane was soaked in 100% methanol prior to transfer and the 1X Tris-Glycine transfer buffer contained 20% methanol. Membranes were blocked for 10 min (Bio-Rad, 12010020), washed in 1× TBST and incubated with the relevant primary antibodies overnight at 4 °C. Membranes were again washed with 1× TBST (5 min, three times), incubated with the relevant secondary antibodies for 1 h at room temperature and protein bands were imaged using ECL substrate (Thermo Fisher Scientific, 32209). All activation and imaging were done using the ChemiDoc XRS+ System (Bio-Rad), while ImageLab version 6.1 was used to analyze Western blots. Target proteins were normalized to total protein using stain-free gel imaging. The different forms of LC3 (LC3I, LC3-II) and OPA1 (L-OPA1/S-OPA1) were distinguished based on molecular weight with reference to the protein ladder. A minimum of *n* = 3 samples were collected for each treatment condition, with original Western blot images for each biological replicate provided in Supplementary File S2. Information about primary and secondary antibodies, including dilutions, is in Supplementary File S1, Table S2.

### 2.6. Mitochondrial Event Localizer Analysis

SH-SY5Y cells were seeded in EMEM:F12 15% FBS at 1 × 10^4^ cells/well in 8-chamber dishes (Thermo Fisher Scientific, 155411PK) and allowed to adhere for 24 h. Subsequently, cells were subject to a 24 h FBS depletion before treatment with 0, 3, 5, and 10 mM PPA for 24 h. A master mix containing 1:1000 TMRE (Thermo Fisher Scientific, T669) and 1:200 Hoechst 33342 (Sigma-Aldrich, H6024) diluted in serum-free media was added to each well 10 min before imaging at the Central Analytical Facility RRID:SCR_023596 (Stellenbosch University, Cape Town, South Africa) using a Carl Zeiss LSM780 AxioObserver Confocal Microscope equipped with Elyra PS1 super-resolution technology, a GaAsP 32+2 PMT detector (ZEISS South Africa, Johannesburg, South Africa) and Zen 2012 Software. Samples were visualized using the LCI Plan-Achromat 63× oil DIC M27 objective (NA = 1.4) (ZEISS South Africa), where TMRE was excited with a DPSS 561-10 laser at 561 nm at 10% intensity and detected at 597–641 nm at a gain of 750, and Hoescht was excited with the Diode 405 nm CW/PS laser at 10% intensity and detected at 410–496 nm at a gain of 900. Each Region of Interest (ROI) was visualized across 10 consecutive time points and 8 z-stacks (z-interval 0.299 μM). A minimum of three regions of interest per condition were visualized and repeated over two independent experiments. Images were analyzed in Fiji ImageJ v1.54p using the previously described Mitochondrial Event Localizer (MEL) pipeline and plugin to quantify fusion and fission events [34]. The MEL pipeline and macro are available at GitHub—rensutheart/MEL-Fiji-Plugin.

### 2.7. Analysis of Mitochondrial Connectivity and Turnover

SH-SY5Y cells were seeded in EMEM:F12 15% FBS at 1.5 × 10^5^ per well on 12 × 0.16 mm glass coverslips (Lasec SA, GLAS2C29M) in 24-well plates, allowed to adhere for 24 h and exposed to a 24 h FBS depletion before treatment. Carbonyl cyanide 4-(trifluoromethoxy) phenylhydrazone (FCCP) (Abcam, Cambridge, UK, ab120081) was used as a positive control for severe mitochondrial dysfunction that could be used to validate subsequent image analysis pipelines. Based on the literature showing a significant increase in mitophagy after a 16 to 24 h exposure to 10 μM FCCP in SH-SY5Y cells [35,36,37], we tested an acute 30 min exposure to 25 μM FCCP, using preliminary plate-based assays to confirm a significant decrease in mitochondrial membrane potential while retaining sufficient cell viability. On the day of staining, all samples were incubated with 300 nM MitoTracker^TM^ Red CMXRos Dye (ThermoFisher Scientific, M7512) for 30 min at 37 °C. Samples were rinsed with 1× phosphate-buffered saline (PBS), fixed with 4% formaldehyde for 20 min and permeabilized with 0.5% Triton-X 100 for 20 min. Subsequently, samples were blocked with 3% BSA for one hour at 4 °C before being incubated overnight with 1:200 monoclonal mouse IgG anti-human LC3-II antibody (ThermoFisher Scientific, MA5-31541) at a dilution of 1:200 in 0.3% BSA at 4 °C. Following two rinses and three five-minute washes in 1× PBS, samples were incubated with 1:1000 Goat anti-Mouse IgG Alexa Fluor Plus 488 (ThermoFisher Scientific, A32723) for 90 min at 4 °C. Lastly, samples were counterstained with 250 nM Dapi (BioLegend, San Diego, CA, USA, 422801) for 10 min, washed and mounted on 26 × 76 mm × 1.2 mm glass microscope slides using 7 μL of Antifade Mountant (ThermoFisher Scientific, S36972) and allowed to dry at room temperature for 2 h before being stored in the dark at 4 °C until imaging.

All samples were visualized using the aforementioned LSM780 AxioObserver Confocal Microscope with the LCI Plan-Achromat 63× oil DIC M27 objective (NA = 1.4). MitoTracker CMX was excited with a DPSS 561-10 laser at 561 nm at 10% intensity and detected at 597–641 nm at a gain of 750, the LC3-II channel was excited with the Argon multiline laser 25 mW at 488 nm and 2% intensity and detected at 499–552 nm at a gain of 750, while Dapi was excited with the Diode 405 nm CW/PS laser at 10% intensity and detected at 410–496 nm at a gain of 800. Each ROI was captured as a series of 0.36 μM slices using unidirectional line sequential scanning, for at least 46 and up to 86 cells per condition or a total of 288 cells from three independent biological repeats. All z-stacks were saved as .czi files, prior to analysis in Fiji/image J according to the pipeline depicted in Figure S1.

All .czi z-stacks were imported into image J using the Bio-Formats Importer plugin and converted into 8-bit .tif files. A maximum intensity projection (MIP) was used to identify individual ROIs each containing one well-segmented cell. Multiplex stacks containing three channels were subject to a subtract background step (rolling ball = 10 pixels), despeckled and smoothed before each channel was separated and pre-processed independently. First, the Iterative Deconvolve plugin was used to perform 10 rounds of deconvolution on each channel. A two-pronged approach was used to quantify mitochondrial content, morphology and connectivity based on the protocol described by Chaudhry et al. (2020) [38]. The MitoTracker channel was despeckled and thresholded using the Li thresholding algorithm. Thresholded 2D slices were analyzed using the Mitochondrial Morphology macro [39] before binarized slices were skeletonized and the “skeletonize 2D/3D” command was used to quantify branches and junctions in the mitochondrial network. Similarly, 3D stacks were used to calculate mitochondrial surface area, volume and integrated density using the “3D object counter” plugin [40]. For the LC3-II channel, the unsharp mask filter was used with a radius = 1 and mask = 0.80 to improve signal:noise ratios, before stacks were binarized using the Li thresholding algorithm and subsequently filtered to remove any particles less than 0.06 μM. LC3-II volume and expression were evaluated using the 3D objects counter. Lastly, colocalization of LC3-II and MitoTracker was measured using the ImageJ JaCoP plugin [40] to calculate Pearsons and Manders colocalization coefficients and associated Costes *p*-values, using a threshold of 2 for both channels, a CCF of 10, and a block size or 8 with 1000 randomizations for each ROI.

### 2.8. Live-Cell Respirometry

Live-cell respirometry was conducted using the Agilent Seahorse XF HS Mini Bioanalyzer (Agilent Technologies, Santa Clara, CA, USA) at the Institute of Infectious Diseases at the University of Cape Town. SH-SY5Y cells were seeded in 6-well plates at a density of 3 × 10^5^ cells/mL in EMEM:F12 with 15% FBS, allowed to adhere overnight and subjected to 24 h of serum depletion before treatment with 5 mM PPA for 72 h. The day before the assay, both treated and untreated cells were seeded in poly-D-lysine (PDL)-coated XFp 6-well miniplates (Agilent Technologies, 103721-100) at 30,000 cells per well and incubated overnight at 37 °C. The next day, media were replaced by 180 μL per well of Seahorse XF DMEM (Aligent Technologies, 103575-100) supplemented with 200 mM glutamine (103579-100), 100 mM pyruvate (103578-100) and 100 mM glucose (103577-100), all at pH 7.4. Seahorse cartridges, having been incubated in MilliQ water at 37 °C overnight, were hydrated with pre-warmed calibrant and both XFp miniplates and cartridges were incubated in a non-CO_2_ 37 °C incubator for 60 min before the assay. Cartridges were loaded according to the protocols detailed by the manufacturers and calibrated for 15 min, while cell media were replaced with fresh XFp DMEM before analysis. MitoStress tests were conducted according to the protocol described by the manufacturer, using sequential injections of targeted mitochondrial electron transport chain (ETC) inhibitors to measure basal, spare and maximal respiratory capacity (Agilent Technologies). Briefly, the assay comprised three cycles of three measurements each at basal respiration, after the addition of 1.5 μM oligomycin, 1.5 μM FCCP and 0.5 μM Rotenone/Antimycin A (Rot/AA). Protein normalization was carried out using the Quick Start^TM^ Bradford protein assay (Biorad, 5000201) according to the manufacturer’s instructions. Absorbance at 595 nM was measured on the Multiskan^TM^ GO microplate reader (Thermo Fisher Scientific), and protein concentrations were calculated from blank-corrected absorbance values according to a standard curve that was run in triplicate with each assay. Lastly, data was exported to the Seahorse Analytics online platform for analysis, ensuring that each run passed quality control checks related to basal respiration, pH, and oxygen availability.

### 2.9. Respiratory Chain Electron Flow Assays

Substrate utilization profiles were characterized using MitoPlate-S1 assays (Biolog, Hayward, CA, USA Cat. #14105) according to the manufacturer’s instructions. Briefly, SH-SY5Y cells were seeded at 2.1 × 10^6^ cells per T75 flask and allowed to adhere for 24 h before a 24 h FBS depletion and a subsequent 72 h exposure to 5 mM PPA. Cells were seeded at a density of 72,000 cells in 30 µL/well and permeabilized with 30 µg/mL Saponin (Sigma-Aldrich, 47036). Absorbance was measured at OD560 for 4 h using a GloMax Explorer (Promega, Madison, WI, USA, GM3500) set to 37 °C. Measurements were taken every 5 min between 30 min and 2 h 30 min, and every 30 min thereafter for a total duration of 4 h. Each treatment was performed across a total of 12 (untreated controls) and eight (5 mM PPA) technical repeats and at least three independent experiments. Slopes, endpoints and Cooks distances were calculated based on the methods described by Radogna et al. (2021) [41] in JupyterLab Notebooks v 7.3.2 using Anaconda Navigator v4.20.0. Metabolites of interest, defined as those showing a significant increase in absorbance between 30 min and 4 h in at least one condition (FDR < 0.0.05), were further assessed using ordinary least squares (OLS) regression, applying MacKinnon and White’s heteroscedasticity-consistent standard errors.

### 2.10. Statistical Analysis

Unless otherwise specified, all data were analyzed in GraphPad Prism v10.0.2 (San Diego, CA, USA). For confocal microscopy data, outliers were removed with the Robust regression and Outlier removal (ROUT) approach using the standard Q coefficient of 1%. All data were tested for normality using the Shapiro–Wilks test before being evaluated for significance. All datasets comprising three or more conditions were analyzed with one-way ANOVAs (parametric) or Kruskal–Wallis (non-parametric) tests with post hoc Dunnet’s or Dunn’s multiple comparisons tests, while MitoStress tests were analyzed using two-tailed paired *t*-tests. *p*-values are annotated on the graphs as follows: * *p* < 0.05, ** *p* < 0.01, *** *p* < 0.001, and **** *p* < 0.0001. Descriptive statistics with the statistical tests and *p*-values associated with each dataset are provided in Supplementary File S3.

## 3. Results

### 3.1. Molecular Disruptions to Mitochondrial Dynamics

To characterize the cytotoxicity of PPA in SH-SY5Y cells, MTT assays were used to measure the relative viability of cells exposed to 3, 5 or 10 mM PPA for 4, 24 and 72 h. Treatment concentrations were informed by previous studies in SH-SY5Y cells, ranging from 0.1–10 mM for 4–72 h [22,23,24,25], as well as plasma concentrations of PPA reported in Propionic Acidemia (PA) that can reach up to 5 mM in fatal cases [14]. We found that PPA (3–10 mM) did not significantly impact cell survival from 4–24 h, while 72 h at 5 and 10 mM decreased cell viability to 64% (± 14) and 27% (± 11) of healthy controls respectively (Figure S2A). This was accompanied by marked changes to gross cell morphology, characterized by elongated cell bodies and spindle-shaped protrusions that emerged after 24 h under PPA stress (Figure S2B).

**Figure 1:**
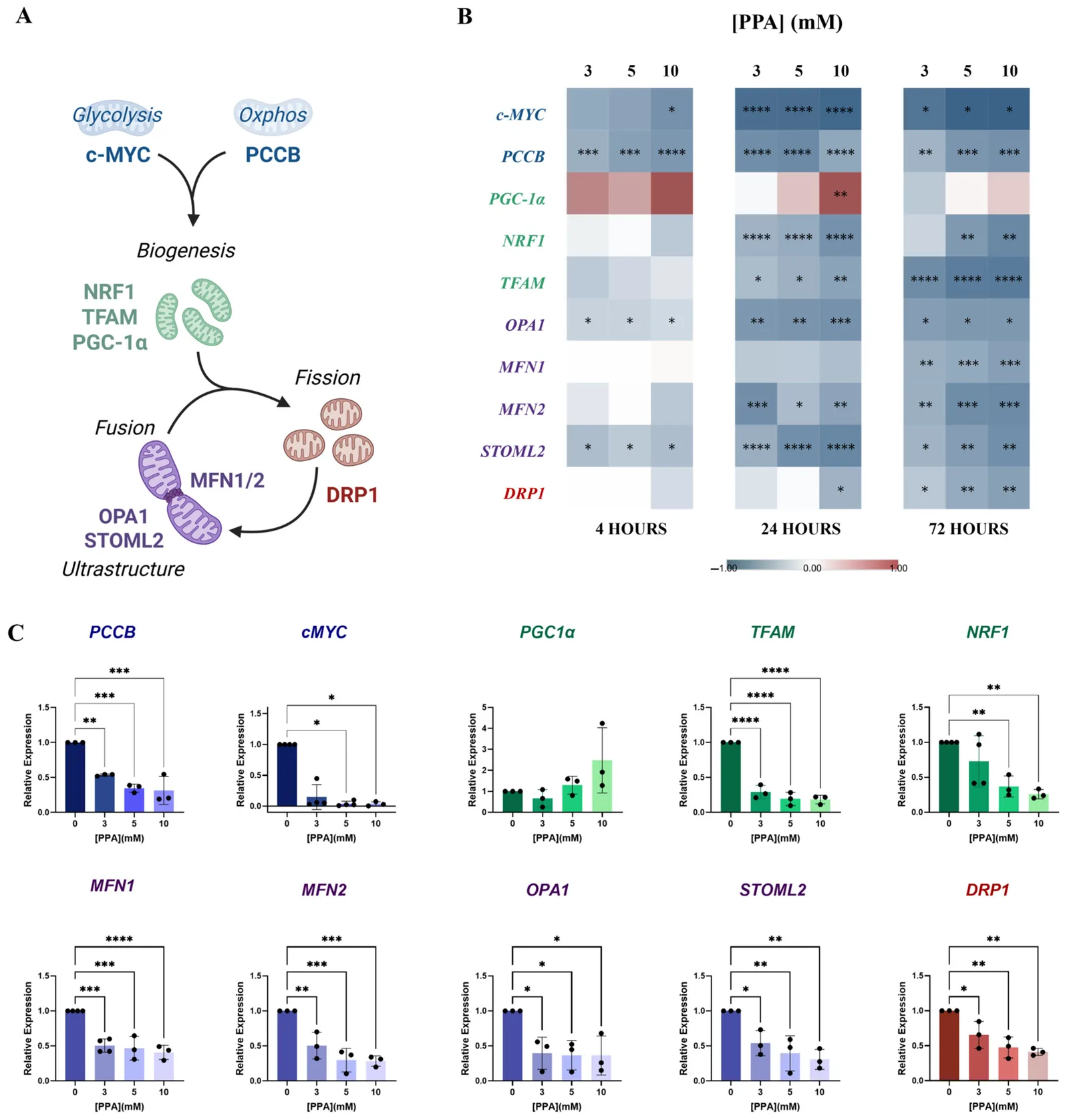
Propionic acid disrupts transcriptional regulators of mitostasis. (**A**) Functional convergence of target genes involved in mitochondrial metabolism (blue), biogenesis (turquoise) and dynamics (purple—fusion; red—fission). Created in Biorender.com. Mahony, C. (2026). https://BioRender.com/a2xm39a. (**B**) Heatmap showing relative mRNA expression of transcriptional regulators in undifferentiated SH-SY5Y cells exposed to propionic acid (PPA) for 4, 24 and 72 h. Data represent the fold change in expression relative to untreated controls according to the ΔΔCT method (2^−ΔΔCT^), where red = upregulated and blue = downregulation. (**C**) Representative scatter plots depicting the mean ± SD (normal distribution) or median ± interquartile range (non-normal distribution) change in expression after 72 h of PPA exposure. All data represent at least *n* = 3 independent experiments performed in triplicate. Data were tested for normality using the Shapiro–Wilks test and significance was established using one-way ANOVAs with Dunnet’s multiple comparisons or Kruskal–Wallis test with Dunn’s Post Hoc Comparisons where * *p* < 0.05, ** *p* < 0.01, *** *p* < 0.001, and **** *p* < 0.0001. PCCB—Propionyl CoA Carboxylase B; c-MYC—c-MYC protooncogene; DRP1—Dynamin-Related Protein 1; PGC1α—Peroxisome Proliferator-Activated Receptor Gamma Coactivator-1 Alpha; NRF1—Nuclear Respiratory Factor 1; TFAM—Mitochondrial DNA Transcription Factor A; MFN1/2—Mitofusin 1/2; OPA1—Optic Atrophy 1; STOML2—Stomatin-Like Protein 2. The arrow depicts the functional convergence of mitostasis, captured in the legend for (**A**). The dots represent the biological repeats described as as *n* = 3 in the legend.

Subsequently, underlying changes to mitochondrial function were evaluated at both nontoxic and cytotoxic doses of PPA. First, the transcriptional responses to PPA were measured using RT-qPCR to study the expression of ten central regulators of mitochondrial metabolism (*PCCB*, *PGC1α*, *c-MYC*), biogenesis (*NRF1*, *TFAM*), fission (*DRP1*) and fusion (*MFN1*, *MFN2*, *STOML2*, *OPA1*) (Figure 1A). Of these, nine genes were significantly downregulated with PPA exposure in a dose- and time-dependent manner (Figure 1B and Figure S3). Notably, we observed an acute and sustained decrease in the expression of *PCCB*, a subunit of propionyl CoA carboxylase that is directly involved in PPA catabolism, where transcription was decreased up to 69% (0.45 ± 0.01 to 0.69 ± 0.20)-fold after 72 h of PPA stress (Figure 1C). Concurrently, PPA altered three interdependent regulators of mitogenesis. While *PGC1α* was transiently upregulated at 24 h, the expression of *c-MYC*, a master regulator of mitochondrial metabolism and biogenesis, was acutely reduced by 78% (±33) after 4 h at 10 mM, reaching a 98% (±7) decrease by 72 h. Both *TFAM* and *NRF1*, two downstream targets of c-MYC-dependent transcriptional regulation, were significantly downregulated only after 24 h, by up to 82% (±6.1) and 74% (±6.7) inhibition after 72 h. Similarly, five genes involved in mitochondrial dynamics were transcriptionally attenuated with PPA exposure. *STOML2* and *OPA1* were significantly reduced after 4 h, reaching a 75% (±4.3) and 68% (±16.7) decrease in expression after 24 h (Figure S3B). On the other hand, a more delayed transcriptional response was observed for *MFN1*, *MFN2* and *DRP1*, which displayed up to 60% (±10.2), 72% (±7.4) and 59% (±5.0) inhibition by 72 h. Together, these data describe a dynamic transcriptional signature associated with PPA exposure that reveals a systemic dysregulation of the molecular mechanisms that govern mitostasis.

**Figure 2:**
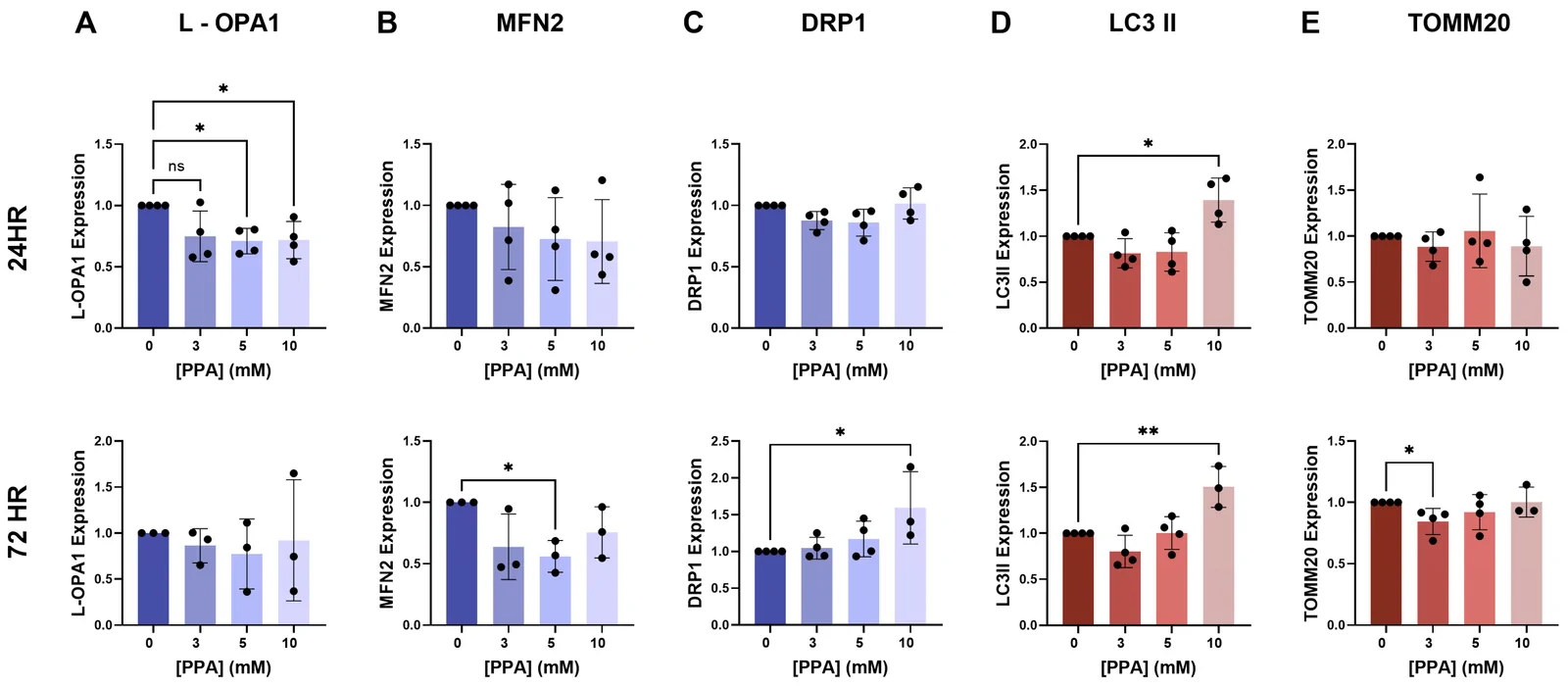
Subtle disruptions to proteins involved in mitochondrial dynamics. SH-SY5Y cells were treated with 3, 5 or 10 mM propionic acid (PPA) for 24 or 72 h before protein extraction and quantification by Western blot. Relative protein expression of (**A**) L-OPA1, (**B**) MFN2, (**C**) DRP1, (**D**) LC3-II and (**E**) TOMM20 where protein abundance was normalized to total protein and fold change in protein expression following was calculated relative to the control (0 mM) for proteins involved in mitochondrial dynamics (purple) and turnover (red). Bars represent mean ± SD or median ± interquartile range for a minimum of *n* = 3 biological repeats. Outliers were removed using the ROUT method and data tested for normality using the Shapiro–Wilks test. Significance was tested using one-way ANOVA with Dunnet’s for multiple comparisons or Kruskal–Wallis using Dunn’s for multiple comparisons; * *p* < 0.05, ** *p* < 0.01, ns = nonsignificant. Original blots for all biological repeats are presented in Supplementary File S2. L-OPA1—Optic Atrophy 1 (long isoform); MFN2—Mitofusin 2; DRP1—Dynamin-Related Protein 1; LC3-II—Microtubule-Associated Protein 1 Light Chain 3 Beta; TOMM20—Translocase of Outer Mitochondrial Membrane 20. The dots represent the biological repeats as detailed in the legend.

Importantly, mitostasis is also modulated by complex post-transcriptional mechanisms that control protein translation, localization and function. Based on the transcriptional profiles presented in Figure 1, we examined a subset of canonical proteins used to study mitochondrial fusion (OPA1, MFN2), fission (DRP1) and turnover (TOMM20, LC3-II) after 24 and 72 h of PPA stress. Here, a clear divergence between transcript and protein expression emerged under the same conditions (Figure 2; corresponding uncropped Western blot images are provided in Supplementary File S2). Although OPA1, MFN2 and DRP1 were transcriptionally downregulated with PPA exposure, protein expression displayed more subtle changes that did not always correlate with gene transcription. Total OPA1 protein levels remained unchanged from 24–72 h (Figure S4A); however, PPA significantly altered the balance between the short (S-OPA1) and long (L-OPA1) isoforms. While S-OPA1 expression remained unchanged (Figure S4B), L-OPA1 was consistently downregulated after 24 h, where expression ranged between 0.7 ± 0.13 and 0.73 ± 0.08 of untreated controls (Figure 2A). A significant decrease in MFN2 was observed only after 72 h at 5 mM (0.62 ± 0.31) (Figure 2B), while DRP1 protein expression was increased 1.59 ± 0.49-fold by prolonged PPA exposure (Figure 2C). LC3-II expression was upregulated at both 24 (1.39 ± 0.24) and 72 (1.47 ± 0.28) h (Figure 2D), while a significant decrease in TOMM20 expression was evident at 24 h (3 mM, 0.89 ± 0.18) (Figure 2E). Ultimately, these data show subtle but distinct disruptions to the proteins that shape mitochondrial homeostasis, which may have functional consequences for mitochondrial dynamics, morphology and function.

### 3.2. Functional Remodeling of Mitochondrial Networks

**Figure 3:**
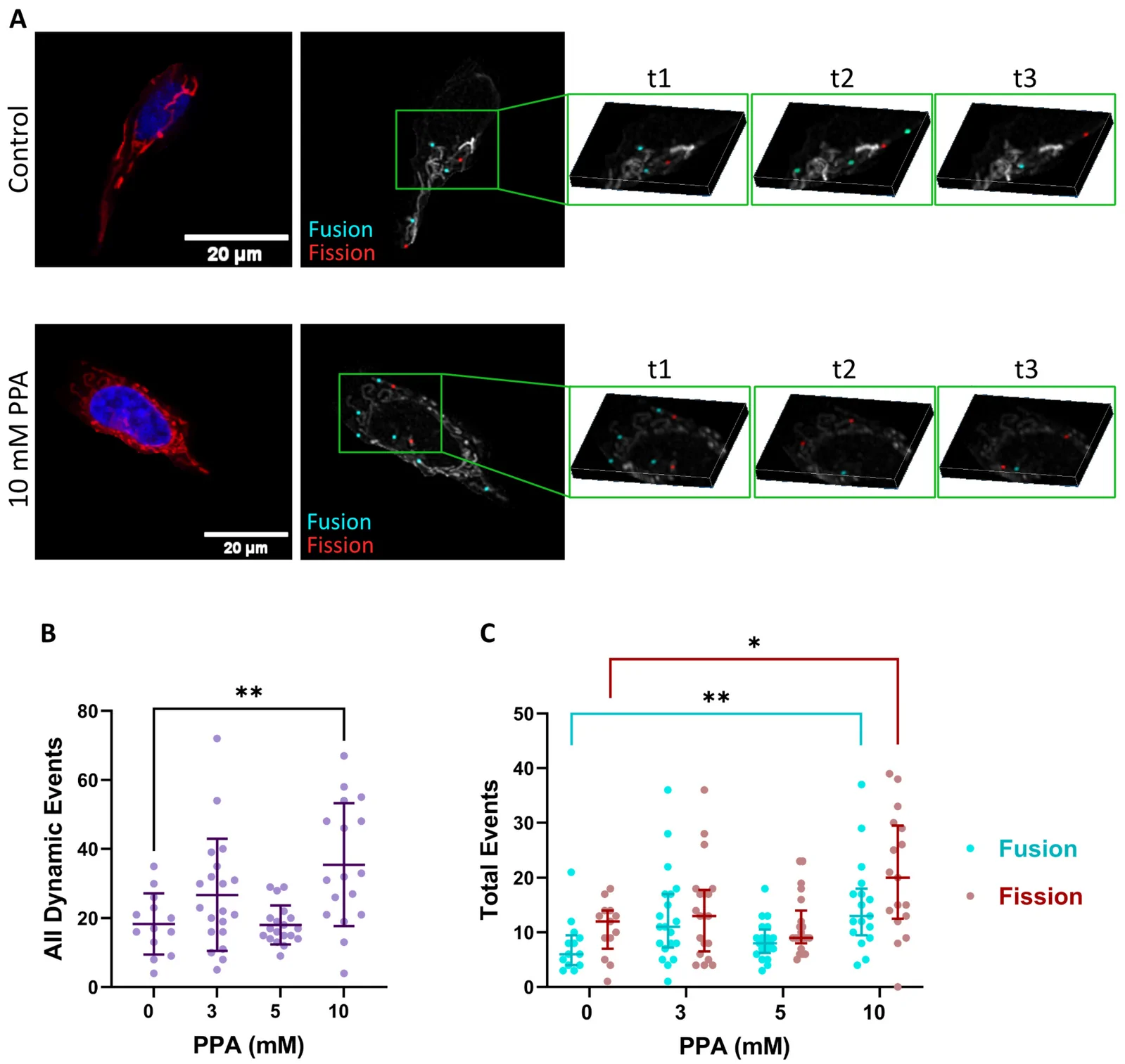
Dynamic mitochondrial remodeling under propionic acid stress. SH-SY5Y cells were treated with 3, 5 or 10 mM propionic acid (PPA) for 24 h before staining with TMRE and Hoechst 33342 for live-cell imaging across nine consecutive time points. (**A**) Representative maximum intensity projections (MIPs) at time frame 2 (t2) and fusion (blue) fission (red) events at time frames 1 (t1) to time frame 3 (t3) for all conditions. Representative MIPs were generated from stacks using the Z-project tool in ImageJ. Representative time frames were generated from the MEL plugin. The MEL plugin in Fiji Image J was used to evaluate the number of (**B**) total dynamic events and (**C**) fusion (blue) and fission (red) events per condition. Data represent mean ± SD for a minimum of *n* = 13 cells per treatment group from two independent biological repeats. Data were filtered using the ROUT method to identify outliers before being tested for normality using the Shapiro–Wilks test. Significance was evaluated using Kruskal–Wallis tests with Dunn’s Post Hoc Comparisons where * *p* < 0.05 and ** *p* < 0.01.

**Figure 4:**
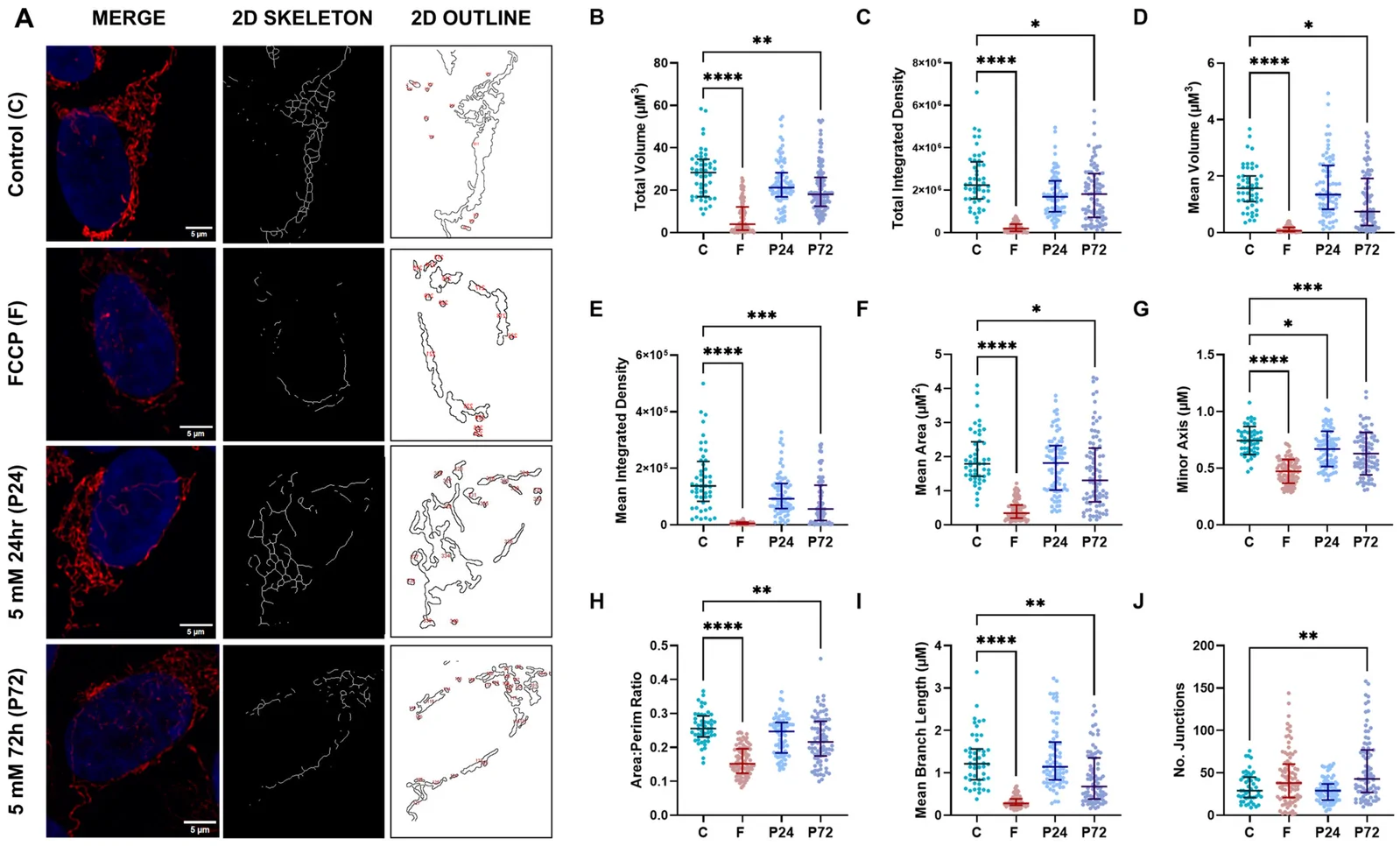
Propionic acid impairs mitochondrial network integrity. (**A**) Representative maximum intensity projections (MIPs), binarized skeletons and 2D outlines of SH-SY5Y cells stained with MitoTracker CMX Ros (Red) and Dapi (blue) with no treatment (C = control), after exposure to 25 μM FCCP for 2 h (F) or 5 mM PPA for 24 (P24) or 72 (P72) hours; scale bar = 5 μM. Cells were visualized with a Zeiss LSM780 Confocal Microscope, using the 63× oil DIC M27 objective with 488 nm laser. Representative MIPs were generated from background-subtracted stacks (rolling ball = 10) using the Z-project tool in ImageJ, and adjusting the display range to 20–140 (Dapi channel) and 0–180 (MitoTracker channel). The 3D objects counter and Mitomorphology plugins in Fiji Image J were used to evaluate (**B**) total mitochondrial volume, (**C**) total integrated density, (**D**) mean mitochondrial volume, (**E**) mean integrated density, (**F**) mean mitochondrial area, (**G**) minor axis and (**H**) area:perimeter ratio for each condition. Binarized skeletons were used to measure (**I**) mean branch length and (**J**) total number of network junctions per cell. Bars represent median ± interquartile range for 288 cells with 46–86 cells per treatment condition from three independent biological repeats. Data was filtered using the ROUT method to identify outliers before being tested for normality using the Shapiro–Wilks test. Significance was evaluated using Kruskal–Wallis test with Dunn’s Post Hoc Comparisons where * *p* < 0.05, ** *p* < 0.01, *** *p* < 0.001, and **** *p* < 0.0001.

To determine whether the molecular changes described above were reflected by downstream changes to mitochondrial function, complementary confocal microscopy techniques were used to characterize mitochondrial network morphology, integrity and dynamics. The treatment paradigm used for functional studies was informed by the cytotoxicity and gene expression profiles presented in Section 3.1. Although 10 mM PPA had the most substantial effect on both transcriptomic profiles and cell viability, 5 mM from 24–72 h was chosen for functional studies with the aim of capturing responses that occur both before and after significant cytotoxicity is observed. Here, the 24 h time point was used to evaluate mitochondrial compensatory responses that may mitigate cytotoxicity, while the 72 h time point was used to study the cytotoxic consequences of prolonged PPA exposure. Time-lapse microscopy in conjunction with MEL analysis revealed dose-dependent changes to mitochondrial dynamics under PPA stress (Figure 3A). We found that high doses of PPA significantly increased the total number of dynamic events (1.93-fold), upregulating the number of both fusion (2.16-fold) and fission (1.76-fold) events after 24 h (Figure 3C). Interestingly, the balance between fission and fusion events was retained under PPA stress (Figure S5), suggesting mitochondrial remodeling that maintains a dynamic state of equilibrium until 24 h. Indeed, confocal microscopy showed that mitochondrial network morphology remained unchanged after 24 h at 5 mM PPA, while signs of network fragmentation emerged after 72 h (Figure 4A). This was illustrated by a significant decrease in total mitochondrial volume and integrated density (Figure 4B,C). In conjunction, PPA significantly reduced mean mitochondrial volume, integrated density and area (Figure 4D–F), decreased mitochondrial width, area:perimeter ratio and mean mitochondrial branch length (Figure 4G–I), and increased the number of network junctions (Figure 4J).

**Figure 5:**
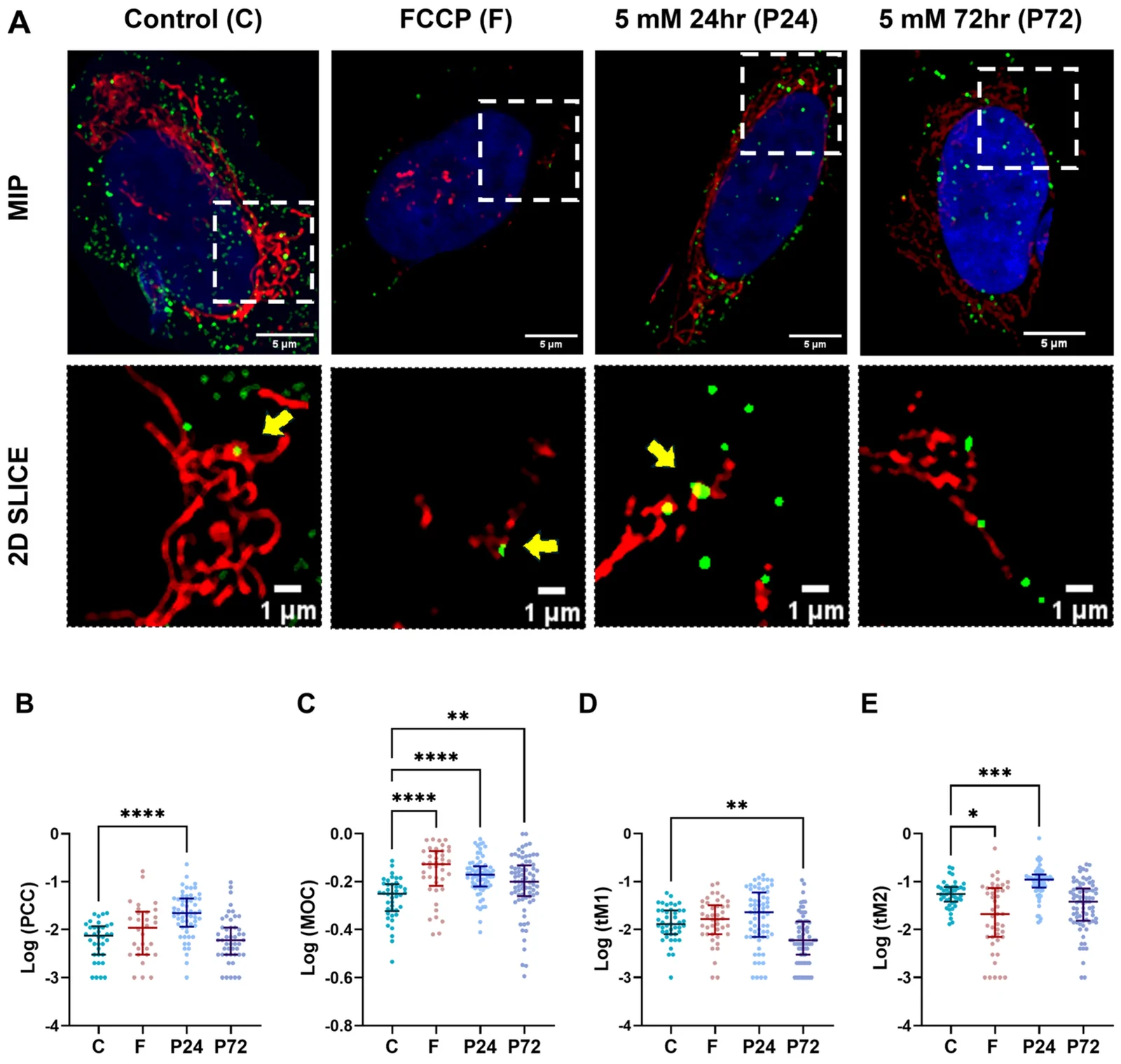
Propionic acid alters mitochondrial turnover. (**A**) Representative maximum intensity projections (MIPs) of SH-SY5Y cells stained with MitoTracker CMX Ros (Red), anti-LC3-II and Alexa Flour488 (green) and Dapi (blue) with no treatment (C = control), after exposure to 25 μM FCCP for 2 h (F) or 5 mM PPA for 24 (P24) or 72 (P72) hours visualized with a Zeiss LSM780 Confocal Microscope, using the 63× oil DIC M27 objective with 488 nm and 561 nm lasers. Representative MIPs were generated from background-subtracted stacks (rolling ball = 10) using the Z-project tool in ImageJ, and adjusting the display range to 20–140 (Dapi channel), 0–180 (MitoTracker channel) or 0–80 (LC3-II channel); scale bar = 5 μM. For each MIP, representative regions of interest (ROIs) from 2D slices are shown, highlighting areas of colocalization with yellow arrows; scale bar = 1 μM. The JaCoP plugin in Fiji/Image J was used to assess colocalization between MitoTracker and LC3-II according to (**B**) Pearson’s correlation coefficient (PCC), (**C**) Manders Overlap Coefficient (MOC) and (**D**,**E**) Manders’ Co-occurrence Coefficients (D—tM1 = MitoTracker and E—tM2 = LC3-II). Bars represent median ± interquartile range for 288 cells with 46–86 cells per treatment condition from three independent biological repeats. Data was filtered using the ROUT method to identify outliers before being tested for normality using the Shapiro–Wilks test. Significance was evaluated using Kruskal–Wallis test with Dunn’s Post Hoc Comparisons where * *p* < 0.05, ** *p* < 0.01, *** *p* < 0.001, and **** *p* < 0.0001.

Next, we examined the colocalization of mitochondrial networks with the autophagosomal protein LC3-II as a preliminary assessment of mitophagic or autophagic flux (Figure 5A). Here, PPA significantly increased the colocalization of LC3-II puncta with MitoTracker CMX after 24 h, illustrated by the increase in the Pearson’s Correlation Coefficient (PCC) (Figure 5B), the Manders Overlap Coefficient (MOC) (Figure 5C) and the LC3-II Manders co-occurrence coefficient (MCC) (Figure 5E). By 72 h, the PCC and LC3-II MCC had normalized, while the MOC remained significantly elevated, possibly reflecting sustained mitophagic activity as both mitochondrial networks and autophagosomes are degraded. Notably, while mean and total mitochondrial volume decreased by 37% and 60% respectively, mitochondrial DNA copy number remained unchanged even under prolonged PPA stress (Figure S6). Indeed, the response to PPA was markedly less severe than the 92–96% reduction in mitochondrial content induced by acute treatment with the ETC uncoupler FCCP. Thus, PPA exposure leads to a dynamic remodeling of mitochondrial dynamics, morphology and clearance that initially appears to maintain mitostasis but subtly erodes mitochondrial integrity over time.

### 3.3. Endpoint Changes to Metabolic State

**Figure 6:**
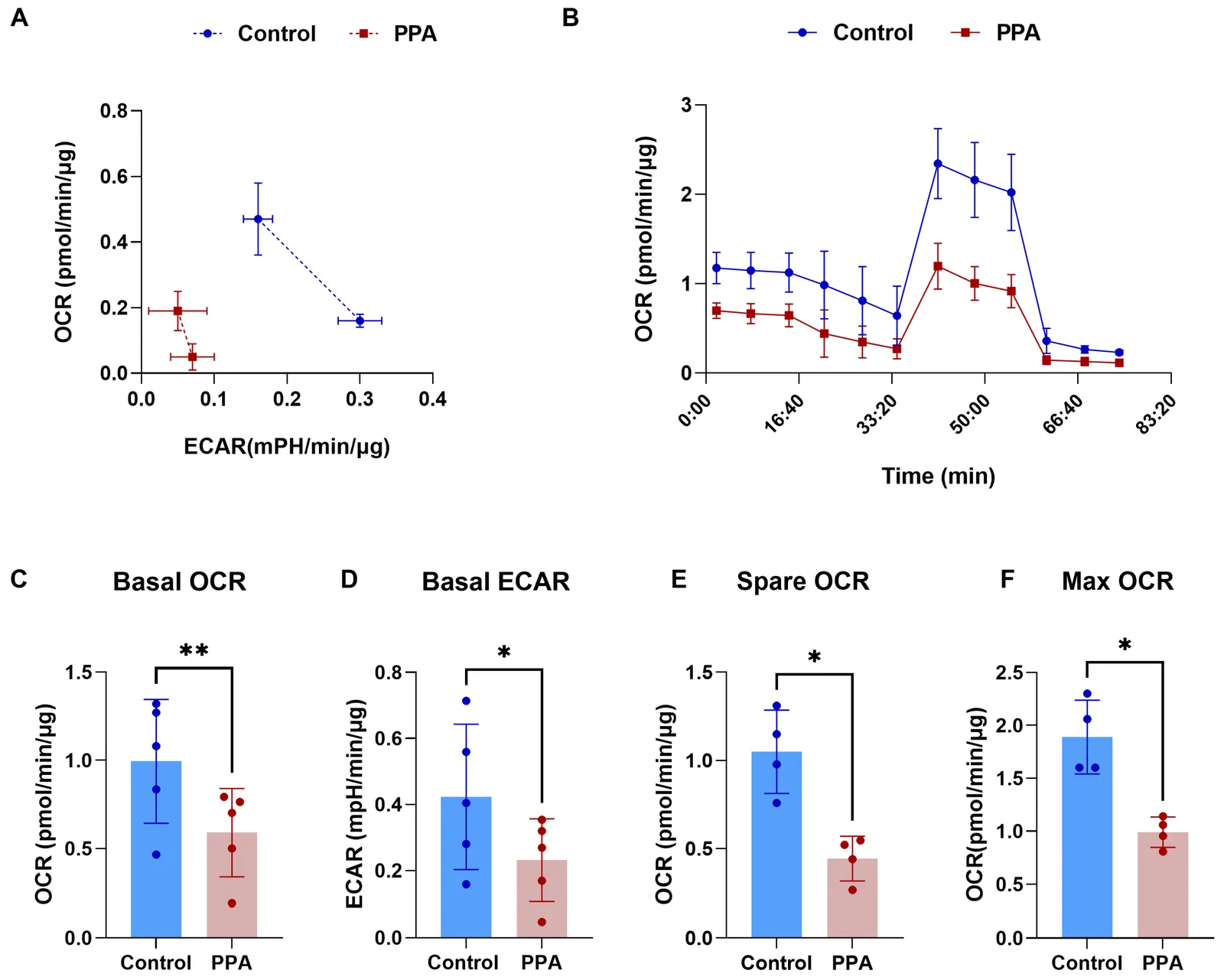
Propionic acid impairs mitochondrial bioenergetics. SH-SY5Y cells were exposed to 5 mM PPA for 72 h before live-cell respirometry was used to assess mitochondrial metabolism. (**A**) The ATP Rate Assay was conducted in triplicate to assess cellular metabolic phenotype (*n* = 1). (**B**) The MitoStress test was used to measure oxygen consumption rate (OCR) and extracellular acidification rate (ECAR) (*n* = 4). (**C**) Basal OCR and (**D**) Basal ECAR measured after three cycles of mixing and measuring. (**E**) Spare respiratory capacity measured as the difference between maximal respiration and basal respiration. (**F**) Maximal OCR calculated as the average of three consecutive cycles after the addition of 1.5 uM FCCP. Data represent the mean ± SD analyzed using paired two-tailed *t*-tests where * *p* < 0.05, ** *p* < 0.01. The dots represent the biological repeats (*n* = 4).

Lastly, we examined whether PPA-induced impairments to mitochondrial dynamics and morphology were reflected by endpoint changes to metabolic state. Metabolic parameters were evaluated after 72 h under 5 mM PPA stress, which corresponds to the time point at which significant changes to mitochondrial integrity were observed with confocal microscopy. First, live-cell respirometry was used to examine PPA-induced disruptions to mitochondrial bioenergetics. Both treated and untreated samples were marked by an oxidative metabolic profile, with more than 90% of ATP production derived from mitochondrial respiration (Figure S7). However, cells exposed to 5 mM PPA for 72 h displayed a distinctly quiescent metabolic phenotype (Figure 6A), characterized by reduced energy consumption and a 60% decrease in the rate of ATP production (Figure S7H). Subsequent MitoStress tests showed that PPA significantly decreased basal OCR and ECAR 0.59- and 0.55-fold respectively (Figure 6B–D), while maximal and spare respiratory capacity were reduced by 48 and 58% (Figure 6E,F). This data illustrates that PPA significantly impairs both oxidative and glycolytic energy metabolism leading to significant deficits in mitochondrial energy production.

**Figure 7:**
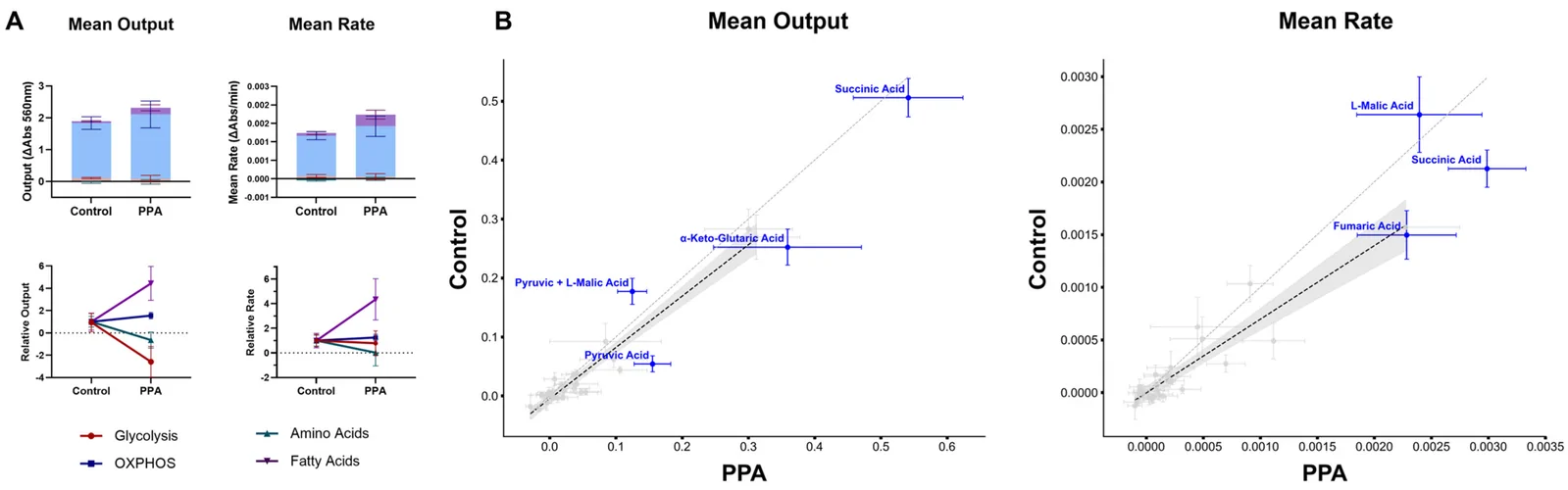
Propionic acid remodels mitochondrial substrate utilization. The Biolog Mitoplate S1 assay was used to assess the utilization of 30 metabolites across four metabolic pathways based on absorbance at 560 nm over a four-hour period. Substrate utilization profiles were characterized using total metabolic output, calculated as the mean change in absorbance at 560 nm between 30 min and 4 h, and mean metabolic rate, calculated as the slope between 30 min and 2 h in delta absorbance at 560 nm per minute. (**A**) Metabolic output and metabolic rate for substrates in all four metabolic pathways in cells exposed to propionic acid (PPA) relative to untreated controls, where data represent mean ± SD (*n* ≥ 3). (**B**) Cooks analysis of total metabolic output and mean metabolic rate for all 30 substrates in control vs. PPA-treated cells identified outlier metabolites (blue) based on a Cooks distance > 4/*n*. The black dashed line represents the linear regression model and the 95% interval, while the light grey line represents a line with the slope = 1.

**Figure 8:**
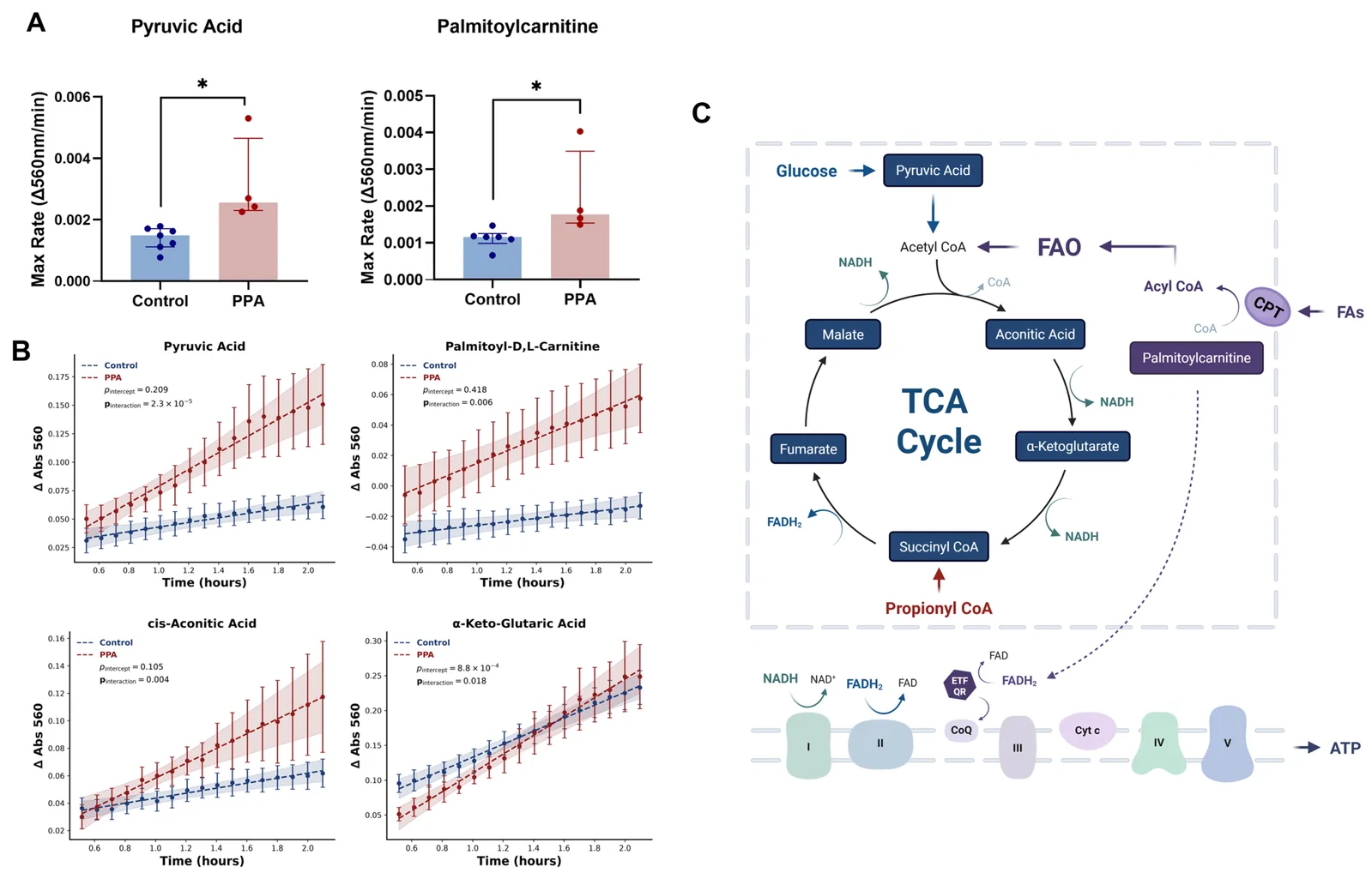
Propionic acid alters TCA cycle flux. Substrate utilization profiles generated from the Biolog Mitoplate S1 assay were assessed for TCA metabolites of interest in SH-SY5Y cells exposed to 5 mM propionic acid (PPA) for 72 h. (**A**) The maximum instantaneous rate of substrate utilization for Pyruvic Acid and Palmitoylcarnitine between 30 min and 2 h where data represent median ± IQR. Data were assessed for normality using the Shapiro–Wilks test and significance was measured using two-tailed Mann–Whitney *t*-tests with BH correction * *p* < 0.05. (**B**) Ordinary Least Squares regressions show the relationship between absorbance at 560 nm and time (between 30 min and 2 h), plotted as y = β0 + β1 (time in hrs) + β2 (condition) + β3 (time × condition) for controls (blue dashed line) and PPA (red dashed line); shading indicates 95% confidence interval. Residuals were assessed for normality using the Shapiro–Wilks test and heteroscedasticity was assessed using the Breusch–Pagan Lagrange Multiplier test. Data points represent the mean ± SEM of at least *n* = 4 independent biological repeats. (**C**) Graphical summary of changes to substrate utilization under PPA stress, highlighting TCA (blue and fatty acid (purple) substrates that are significantly altered by PPA. Created in Biorender.com. Mahony, C (2026). https://BioRender.com/fyofbeo.

Furthermore, substrate utilization profiles suggested significant changes to TCA cycle flux under PPA stress. Of the 31 metabolites measured using the Biolog Mitoplate S1, TCA substrates accounted for more than 95% of metabolic output under both conditions (Figure 7A). Ten metabolites of interest contributed significantly to metabolic output in at least one condition, eight of which were TCA intermediates (FDR < 0.05; Table S3). Again, this demonstrates a level of oxidative reliance consistent with a neuronal phenotype. Interestingly, both the mean and maximal rate of substrate utilization appeared to be increased under PPA stress, driven by changes to TCA and fatty acid metabolism (Figure 7A). Based on both total metabolic output and mean metabolic rate, Cook’s distances demonstrated an increased utilization of six TCA substrates that directly produce reducing equivalents for the ETC—including pyruvate, α-ketoglutarate, succinate and malate (Figure 7B). Interestingly, PPA more than doubled the maximum utilization of pyruvic acid, which bridges cytoplasmic glycolysis to oxidative phosphorylation, and palmitoylcarnitine, which links fatty acid B-oxidation to TCA flux (Figure 8A; Table S4). Moreover, OSL regression analyses established significant changes to the utilization profiles of both pyruvic acid and palmitoyl carnitine, as well as two other early TCA intermediates—aconitic acid and α-ketoglutarate (Figure 8B; Table S5). Together, this data suggests distinct disruptions to TCA anaplerosis linked to carbon and fatty acid catabolism under PPA stress (Figure 8C).

## 4. Discussion

The neurotoxic effects of PPA are widely documented in the preclinical and clinical literature; however, the underlying pathophysiology is not mechanistically understood. Here, we used the SH-SY5Y cell line to study the molecular, morphological and metabolic signatures of PPA-induced neurotoxicity. Several studies have harnessed the flexible nature of undifferentiated SH-SY5Y cells to study PPA stress [21,22,23,24,25]; however, few have considered the context-specific utility of this system to examine mitochondrial phenotypes. The SH-SY5Y system displays an oncogenic metabolic profile that facilitates cell-line expansion but deviates significantly from the neuronal phenotype. Unlike the intrinsic oxidative reliance of primary neurons, SH-SY5Y cells display an “opportunistic” metabolic profile that shifts with nutrient availability [42] and a reciprocal relationship between oxidative and glycolytic metabolism [43] that allows them to compensate for mitochondrial dysfunction [44,45,46]. Still, undifferentiated SH-SY5Y cells display a degree of oxidative reliance that surpasses non-neuronal cell lines even if oxidative capacity is more limited than in mature neurons [47,48]. Importantly, Naia et al. [30] demonstrated that an oxidative phenotype could be promoted by replacing glucose with alternative carbon sources, while Kronenberger et al. [31] showed that 24 h of serum depletion significantly reduced the glycolytic reliance of SH-SY5Y cells. Thus, we used low-glucose EMEM:F12 with glutamine supplementation and serum depletion to promote an oxidative metabolic landscape within which to characterize PPA stress.

First, we established the cytotoxicity of PPA stress in SH-SY5Y cells cultured under these conditions. While cell viability remained unchanged from 4 to 24 h, significant cell death was observed after 72 h at both 5 and 10 mM. Interestingly, previous studies in DMEM:F12 reported significant cytotoxicity at 1, 5 and 10 mM PPA after 24 [22,25], 48 and 72 h [24]. However, like our study, similar experiments using EMEM:F12 found no changes to cell viability after 24 h at doses of up to 10 mM PPA [23]. This discrepancy underlines the need to consider how basal metabolic state influences studies of mitochondrial dysfunction in the SH-SY5Y model, suggesting differential responses under stress that warrant further investigation. Our data highlighted a window from 24 to 72 h at 5–10 mM PPA that captured the earliest shift from mitochondrial compensation to acute dysfunction in our cell model system.

In a clinical context, circulating levels of PPA range between 0.4 and 6 μM, while concentrations between 10 and 30 mM have been reported in the human colon [49,50]. The doses sufficient to induce cytotoxicity in vitro represent supraphysiological concentrations observed in severe cases of PA where circulating propionate can reach between 4 and 5 mM [14,51]. These doses are consistent with the established PPA murine models for ASD, which administer 500 mg/kg or 26 mM for up to 5 days [10,18,52,53]. The treatment paradigms used in preclinical models do not necessarily reflect the chronic pathophysiological conditions associated with PA and other metabolic diseases in a clinical context. Rather, each model system can provide insight into mechanisms that may drive mitochondrial dysfunction in response to acute metabolic stress. Previous studies in the SH-SY5Y cell line have documented signs of mitochondrial dysfunction emerging after 24–72 h at 1–10 mM PPA [22,24]. Here, we report a dose-dependent decrease in cell viability above 5 mM from 24 to 72 h. Accordingly, this window was used to inform subsequent mechanistic studies of mitochondrial dysfunction. The molecular regulation of mitostasis is governed by a complex interplay between transcriptional and post-transcriptional mechanisms that shape mitochondrial dynamics. Here, we examined the mRNA and protein expression profiles of canonical mitochondrial genes involved in four central aspects of mitostasis. We found that PPA significantly disrupted transcriptional regulators of mitochondrial metabolism (*PCCB*, *PGC1α*, *c-MYC*), biogenesis (*TFAM*, *NRF1*), fusion (*MFN1*, *MFN2*, *OPA1*, *STOML2*) and fission (*DRP1*) in a dose- and time-dependent manner. As a subunit of propionyl CoA carboxylase, *PCCB* is directly involved in PPA catabolism; indeed, PPA is commonly used to model PCC dysfunction. Yet, the relationship between PPA and the PCC gene itself remains poorly characterized. We found that PPA immediately reduced *PCCB* expression by up to 55%, which was sustained throughout the 72 h exposure. This reveals a novel relationship between PPA exposure and *PCCB* expression that remains understudied in the PPA model. Importantly, the correlation between mRNA transcription, protein expression and enzyme activity is complex. Studies have found that *PCCB* protein levels are better predicted by *PCCA,* rather than *PCCB*, mRNA expression, as the *PCCB* monomer is stabilized in complex with PCCA [54,55]. Moreover, both PCCA and *PCCB* proteins are post-translationally cleaved by mitochondrial matrix proteases, and the function of the PCC enzyme is biotin-dependent [56]. Nevertheless, it is worth noting that in vitro studies have revealed an essential role for *PCCB* as a chaperonin during PCC complex assembly [57,58,59], while data from PA patient fibroblasts demonstrated a correlation between *PCCB* mRNA and PCC function [57]. A comprehensive assessment of the transcription, translation, modification and localization of the PCCA and *PCCB* subunits would be needed to demonstrate an undisputed relationship between PPA and PCC function. Here, the administration of PPA reflects a biochemical consequence of PCC dysfunction that may reflect disruptions to mitochondrial function.

In fact, PPA exposure induced significant disruptions to both *c-MYC* and *PGC1α*, each of which function as master regulators of mitochondrial metabolism and biogenesis [60]. Of the ten mitochondrial genes measured in this study, *c-MYC* was the most substantially downregulated across all twelve conditions, reaching more than 90% inhibition by 72 h. Markedly, *c-MYC* modulates the transcription of 599 mitochondrial genes required for mitochondrial biosynthesis, assembly and function [61] and a reciprocal relationship between *c-MYC* and mitochondrial dysfunction is well-documented in the literature [62,63,64,65]. Graves et al. (2012) [64] showed that *c-MYC* knockdown decreased mitochondrial content, polarization, size, cristae complexity and connectivity, impaired oxidative and glycolytic metabolism and altered the expression of key fission and fusion proteins including OPA1, MFN1, MFN2 and DRP1. Conversely, metabolic stress has been found to decrease *c-MYC* expression in SH-SY5Y cells [65]. Notably, the downregulation of *c-MYC* induced by metabolic stress was found to promote mitochondrial fission and apoptosis [66], while inhibiting *c-MYC* was sufficient to attenuate stress-induced apoptosis in vitro [63]. It remains unclear whether the transcriptional suppression of *c-MYC* observed in our model reflects a cause, or consequence, of mitochondrial dysfunction. However, the significant and sustained downregulation of *c-MYC* represents a relevant target for future mechanistic studies into PPA-induced neurotoxicity.

Notably, several studies have shown that suppressing *c-MYC* decreased the expression of its targets *TFAM* and *NRF1*, leading to impaired mitochondrial respiration and increased oxidative stress [67,68,69,70]. Here, the decrease in *c-MYC* expression was reflected by a significant downregulation of both *TFAM* and *NRF1* from 24 to 72 h of PPA stress, which may reflect the role played by *c-MYC* as one of the upstream transcriptional regulators of mitogenesis. Kim et al. (2019) previously reported that PPA decreased *TFAM* mRNA expression after 4 h in SH-SY5Y cells [22], while studies in colorectal carcinoma found that both *TFAM* and *NRF1* were downregulated with long-term PPA exposure [71]. Interestingly, Kim et al. (2019) found that TFAM protein expression increased under the same conditions [22], while Tang et al. (2011) revealed a pattern of acute upregulation followed by transcriptional inhibition over time [71]. Moreover, several studies have found that PPA increased mitochondrial biogenesis by upregulating *TFAM* or *NRF1* in preclinical models [22,72,73,74,75]. Thus, the transcriptional mechanisms that mediate PPA-induced disruptions to mitogenesis appear to be multifaceted, dynamic and context-dependent and may not always reflect downstream changes to protein expression and function.

This was exemplified by the transcriptional response of *PGC1α*, which juxtaposed the pattern of dose-dependent downregulation displayed by the other genes of interest. Rather, *PGC1α* displayed varying patterns of upregulation from 4 to 24 h of PPA stress, with evidence for significant dysregulation at 24 h. This is consistent with previous studies in SH-SY5Y cells, which reported a 7.5-fold increase in *PGC1α* mRNA and protein expression after 4 h at 5 mM PPA [22]. Similarly, 2–4 mM PPA upregulated *PGC1α* transcription in bovine enterocytes [76] and increased mRNA and protein expression in bovine-derived hepatocytes [74,77]. Interestingly, *PGC1α* expression was also increased 1.5-fold in *PCCB*-/- iPSC-derived cardiomyocytes compared to the isogenic control [78]. As an energy-sensitive regulator of mitostasis, *PGC1α* responds to ATP depletion, redox state or intracellular Ca2+ flux via the AMPK, cAMP, NAD+/SIRT1, or CaMK pathways respectively [79]. Thus, the variability in *PGC1α* expression under PPA stress may be due to complex and interdependent mechanisms that govern *PGC1α* expression in response to metabolic and oxidative state. The increase in *PGC1α* expression observed here, while highly variable, reflects previous models of PPA stress and *PCCB* dysfunction and may suggest disruptions to energy-sensitive signaling pathways that modulate transcription.

Notably, *PGC1α* has been shown to modulate the transcription and activity of *MFN1*/2, *DRP1* and *OPA1* [80], thereby coupling the regulation of mitochondrial biogenesis, fission and fusion. Under stress, fusion serves as a form of complementation by integrating dysfunctional mitochondrial components into functioning organelles, while fission facilitates the removal and clearance of damaged units in the mitochondrial network [81]. Mitochondrial network integrity is maintained by fission and fusion events driven by interactions between *STOML2* and the dynamin-related GTPases *MFN1*, *MFN2*, *OPA1* and *DRP1* [82]. While metabolic stress has been shown to disrupt *OPA1*, *MFN1/2* and *DRP1* transcription in SH-SY5Y cells [83,84,85], the genes involved in mitochondrial dynamics remain uncharacterized in the PPA model. Here, transcriptional signatures revealed a coordinated downregulation of fusion and fission machinery in response to PPA.

*OPA1* and *STOML2* were the most significantly impacted by PPA stress, displaying convergent transcriptional profiles marked by a 70% decrease in expression by 24 h at 10 mM. Interestingly, *OPA1* and *STOML2* interact directly to remodel mitochondrial ultrastructure by facilitating the assembly of respiratory super-complexes in the mitochondrial membrane [86,87,88]. In fact, *STOML2* also modulates mitochondrial biogenesis, fission, fusion and mitophagy via interactions with cardiolipins and prohibitins [89], *MFN2* [90], *PINK1* and PARK [91,92,93,94] respectively. In this context, it is worth noting recent work from our group that revealed significant disruptions to mitochondrial morphology and ultrastructure under PPA stress [26], which is supported by similar findings from independent studies in SH-SY5Y cells [22,24] and PA-derived hepatocytes [95]. The transcriptional inhibition of *OPA1* and *STOML2* observed here may propose a mechanism that precedes downstream disruptions to mitochondrial ultrastructure, which could significantly influence mitochondrial integrity and respiratory capacity under stress.

Collectively, the transcriptional profiles of PPA exposure suggest an erosion of the adaptive capacity maintained by fission and fusion to mitigate mitochondrial dysfunction under stress. Importantly, the transcriptional profiles of individual genes may not reflect acute changes to specific mitochondrial processes. Fission, fusion or biogenesis may be induced as an acute response to metabolic stress by activating or recruiting existing transcripts and proteins. The data presented here cannot necessarily distinguish general intracellular stress responses from those induced specifically by PPA, although both provide insight into PPA-associated toxicity. Moreover, further work will be required to identify the mechanisms of transcriptional regulation that alter gene expression under PPA stress—and to characterize downstream changes to protein translation, modification and activity that directly alter mitochondrial function.

Accordingly, we examined the expression profiles of select proteins involved in mitochondrial quality control. As well as the canonical GTPases that govern mitochondrial dynamics (MFN1/2, OPA1 and DRP1), TOMM20 and LC3-II were assessed as a measure of mitochondrial turnover. Interestingly, none of the genes examined displayed a linear relationship between mRNA and protein expression under PPA stress. Indeed, the proteins involved in mitochondrial dynamics remained largely unchanged despite the transcriptional downregulation observed under the same conditions. This is consistent with our previous data showing divergent mRNA and protein expression profiles under PPA stress [26]. Similarly, Kim et al. (2019) previously differential responses to transcript and protein expression under PPA stress [22]. Additional work will be required to understand this phenomenon, which may yield novel insight into PPA-induced mitochondrial dysfunction. Despite the discrepancy between the transcript and protein expression of individual genes, both datasets reflect convergent disruptions to mitochondrial dynamics.

We found that PPA significantly reduced the expression of MFN2 and L-OPA1 proteins, while increasing total DRP1 and LC3-II expression at high doses. In particular, PPA altered the balance between the long and short OPA1 isoforms that govern mitochondrial connectivity, content and ultrastructure. L-OPA1 has been shown to be necessary for both mitochondrial fusion and cristae organization, while a shift towards the S isoform promotes mitochondrial fission [96]. Moreover, we showed that PPA upregulates the DRP1 GTPase that has been shown to drive mitochondrial fission, mitophagy and cell death in SH-SY5Y cells exposed to metabolic and oxidative stress [46,84,97,98]. This is consistent with the observed increase in LC3-II, an essential component of the autophagosomes that are recruited to damaged mitochondria tagged for degradation [99]. The decreased L-OPA1:S-OPA1 ratio with the increase in both DRP1 and LC3-II expression may reflect a shift towards mitochondrial fission and fragmentation that is characteristic of metabolic stress. Importantly, future studies should assess the mitochondrial localization of proteins of interest to more specifically link changes in protein expression to mitochondrial morphology and dynamics. Nevertheless, these data suggest a shift in the balance between fission and fusion proteins that may alter mitochondrial dynamics and turnover under PPA stress.

This observation was further supported by quantitative measures of mitochondrial morphology and dynamics. Time-lapse microscopy revealed a significant increase in the number of both fission and fusion events under PPA stress. Here, the balance between fission and fusion events was retained even as dynamic events increased in frequency, indicating a remodeling of mitochondrial networks to maintain mitostasis. In conjunction, we observed a significant upregulation of mitochondrial remodeling that retained mitochondrial integrity after 24 h. However, prolonged PPA exposure appeared to overshoot this capacity for adaptive remodeling, culminating in mitochondrial network fragmentation and an erosion of mitochondrial content by 72 h. Unlike the global degradation of the mitochondrial network induced by FCCP, we found that PPA significantly reduced mitochondrial connectivity and content without a concurrent decrease in either mtDNA copy number or mitochondrial count. While mtDNA copy is less sensitive to changes in mitochondrial mass, both TOMM20 and MitoTracker reflected a comparatively subtle remodeling of mitochondrial dynamics that sustained mitostasis to a point and better retained mitochondrial content even as the balance between stress and compensation was disrupted.

Notably, the fragmented network morphology that emerged with prolonged PPA exposure is also characteristic of cells derived from PA patients [95] and reflects prior studies of PPA administration in vitro [100]. However, the mechanisms that alter mitochondrial network integrity under PPA stress remain under investigation. Recent studies have shown that toxic propionyl derivatives disrupt mitochondrial morphology by inhibiting the MICOS complex [75], modulating mitochondria-endoplasmic reticulum (ER) contacts [101] or increasing DRP1 protein expression and oligomerization [102,103]. Likewise, Jung et al. (2020) reported an upregulation of mitophagy in SH-SY5Y cells exposed to PPA (1–2 mM) after 24 h [24], while Schumann et al. (2021) reported impaired mitophagic flux in PA-derived cell lines [95]. Indeed, PPA has been found to upregulate autophagy in hippocampal neurons [17,104] as well as other cancer-derived, bovine and murine cell models [23,71,105,106,107,108,109] with several studies proposing a role for autophagy as a protective mechanism to mitigate PPA-induced apoptosis in vitro.

We observed an increased colocalization between LC3-II and mitochondrial networks after 24 h of PPA stress, which may suggest changes to mitophagic or autophagic flux. Importantly, LC3-II is detected upon the formation of mature autophagosomes but not after their subsequent degradation in autolysosomes. Thus, an increase in colocalization may reflect either an increase in mitophagic flux or impairments to autophagosomal clearance. Thus, the method used here cannot be used as a measure of total autophagic activity, nor to discriminate between macromitophagy and micromitophagy that differentially contribute to mitochondrial quality control in a cell-specific manner. It should also be noted that serum depletion may have influenced stress-induced autophagic responses in our system. Additional measures of autophagy (LC3α, p62, or autolysosome inhibitors) or mitophagy (PINK1 or PARKIN) would be required to mechanistically characterize how PPA alters mitophagic activity. The data presented here demonstrate only that PPA appears to perturb the balance between autophagosome formation and clearance relative to untreated controls, which contextualizes the associated changes to mitochondrial morphology.

Notably, these morphological changes were reflected on the functional level, where PPA impaired oxidative and glycolytic energy production by reshaping metabolic flux. Both live-cell respirometry and substrate utilization profiles revealed an oxidative metabolic profile in our SH-SY5Y model, with a mitochondrial reliance above 90%. While SH-SY5Y cells grown in high-glucose, FBS-rich medium typically display a preference for glycolysis [31], our model reflects the degree of oxidative reliance previously reported in primary neurons and glucose-deprived SH-SY5Y cells [30,110]. Nevertheless, our data still revealed a capacity to upregulate glycolysis in response to mitochondrial inhibition in the absence of PPA stress. On the other hand, 72 h at 5 mM PPA significantly suppressed mitochondrial respiration, depleted spare respiratory capacity and inhibited both basal and compensatory glycolysis. As a result, PPA decreased total ATP synthesis by more than 60%, while basal, spare, maximal and non-mitochondrial respiration were impaired by at least 40%. In contrast to the targeted mitochondrial dysfunction induced by ETC inhibitors, this suggests a distinct feature of PPA stress that not only impedes oxidative respiration but simultaneously attenuates the capacity for glycolytic compensation in SH-SY5Y cells.

Interestingly, substrate utilization profiles may yield insight into the mechanisms underlying this response. Despite the significantly reduced mitochondrial activity measured using live-cell respirometry, PPA exposure systemically increased the utilization of TCA cycle intermediates in MitoPlate S1 assays. This apparent discrepancy is mechanistically consistent with the functional outputs obtained in each assay, considering the underlying differences between the two experimental systems. In each case, cells were pretreated with 5 mM for 72 h before metabolic state was assessed under standardized conditions optimized for the assay in question. Live-cell respirometry uses intact cell cultures supplied with DMEM containing glucose, glutamine and pyruvate. This approach accounts for the coupling between cytosolic and mitochondrial reactions that drive both glycolytic and oxidative energy production. On the other hand, the MitoPlate system bypasses cytoplasmic metabolism entirely, using saponin to selectively permeabilize cholesterol-rich cell membranes while leaving mitochondrial membranes intact [41]. Here, permeabilized cells are suspended in a specialized isotonic medium providing single metabolites in isolation rather than the complete DMEM used for respirometry. The permeabilization of cell membranes effectively abolishes cytoplasmic glucose metabolism, due to the leakage of essential enzymatic cofactors such as NAD+ and NADP+ that govern metabolic and oxidative state [111]. Hence, any inhibitory effect on mitochondrial bioenergetics mediated by cytosolic factors—such as glycolytic flux, free metabolites, or redox ratios—may be alleviated under these conditions. The functional readout of substrate utilization in this context is, more precisely, a measure of how efficiently mitochondria are able to use individual metabolites once endogenous cytosolic regulation is attenuated.

We observed that cells pretreated with PPA more effectively oxidized TCA metabolites coupled to the reduction of NADH (pyruvate, aconitate, α-ketoglutarate, malate) and FADH (succinate, fumarate) linked to ETC complex 1 and complex 2 activity respectively. In particular, PPA more than doubled the rate of pyruvate utilization, while disrupting the flux of both α-ketoglutarate and succinate, where anaplerosis integrates propionyl CoA into the TCA cycle. Concurrently, PPA increased the utilization of palmitoylcarnitine, which functions as an important intermediate involved in the transport and oxidation of long-chain fatty acids via the carnitine shuttle [112]. Importantly, while palmitoylcarnitine can be oxidized in the TCA cycle, it can also be metabolized directly via the electron transfer flavoprotein quinone oxidoreductase (ETF/QOR) pathway that drives the reduction of coenzyme Q, or ETC complex 3 [113]. Moreover, fatty acid B-oxidation is highly sensitive to the free CoA, carnitine and redox ratios in both the cytosol and the mitochondrial matrix. Thus, the substrate utilization profile associated with PPA stress reveals a metabolic priming of reactions used to deliver reducing equivalents to the mitochondrial ETC when the inhibitory effect of cytosolic regulation is alleviated. This data suggests that the bioenergetic deficits measured using live-cell respirometry may be due not to functional deficits in the mitochondrial ETC itself, but that disruptions to free metabolite ratios impede the glycolytic and oxidative reactions that fuel energy production.

These data are consistent with prior studies documenting the biochemical signature associated with PPA exposure in published model systems. It is well-established that toxic levels of PPA can lead to an accumulation of several metabolic byproducts that disrupt oxidative phosphorylation (OXPHOS) via both direct and indirect mechanisms. Propionyl CoA directly inhibits several TCA cycle enzymes, including pyruvate dehydrogenase (PDH), alpha-ketoglutarate dehydrogenase (αKGDH) and succinate CoA ligase (SUCL), leading to decreased pyruvate, αKG and succinate oxidation, reduced ETC activity and diminished ATP and GTP production [55]. As propionyl CoA accumulates, there is also an increase in the production of succinyl CoA and methylcitrate, which inhibits both citrate synthase (CS) and αKGDH [114,115]. At the same time, elevated acyl CoA:CoA ratios inhibit the terminal stage of glycolysis mediated by pyruvate kinase (PK) [116], decreasing the production and oxidation of pyruvate as an energy source.

Previous work has also shown that both propionyl and succinyl CoA broadly disrupt mitochondrial CoA metabolism by directly sequestering CoA, inhibiting CoA synthesis and dysregulating the mechanisms that maintain CoA homeostasis [117]. PPA perturbs the balance between fatty acid import, export and sequestration in the mitochondrial matrix, depleting both the free carnitine required for fatty acid import, and the free CoA that facilitates OXPHOS and B-oxidation [118]. Interestingly, PPA administration has been shown to decrease free CoA in vitro [16,119], while upregulating CoA biosynthesis was able to alleviate the metabolic deficits associated with PCC dysfunction in vivo [120,121]. Thus, CoA depletion may be a common mechanism that underpins the changes to glucose and fatty acid metabolism observed in the PPA model. In fact, the CoA depletion hypothesis may explain the synergistic inhibition of both glycolytic and oxidative ATP production despite the increased utilization of TCA intermediates we observed under PPA stress. A decrease in cytosolic CoA could disrupt the supply of pyruvate and acetyl CoA to the TCA cycle by inhibiting glycolytic flux and fatty acid import, corresponding to the impaired oxidative and glycolytic ATP production measured in live cells. However, cytosolic acyl CoA:CoA imbalances would be negated by permeabilizing cell membranes. The increased efficiency of pyruvate and plamitoylcarnitine utilization under these conditions may suggest a compensatory upregulation of enzyme activity that serves to mitigate the inhibitory effects of PPA on TCA flux. While the mechanisms driving metabolic remodeling and compensation under PPA stress require further elucidation, it is worth noting the functional implications for neuronal function. Both glucose and fatty acid metabolism are tightly regulated during neurogenesis to facilitate the production of sterols, glycoconjugates, and phospholipids that form neuronal membranes, extracellular matrix components and synaptic vesicles [122]. Thus, PPA-induced metabolic reprogramming may highlight a neurotoxic mechanism that contributes to the neurological component of PA and other inherited metabolic diseases.

Interestingly, substrate utilization profiles may also be linked to the morphological remodeling induced by PPA. A reciprocal relationship between metabolic state and mitochondrial dynamics is well-documented [28] and several studies have shown that succinate, pyruvate and short-chain fatty acids differentially impact mitochondrial elongation and connectivity [123,124]. Changes to mitochondrial FAO have been found to modulate mitochondrial fragmentation by shifting the balance between fatty-acyl CoA import and export [125]. Moreover, the inhibitory effect of PPA on malonyl CoA synthesis may have implications for mitochondrial fatty acid synthesis (mtFAS), which governs mitochondrial volume, morphology, dynamics and ultrastructure by modulating the composition of the mitochondrial membrane [126]. Interestingly, a loss of ΔΨm associated with TCA inhibition is known to induce mitochondrial fragmentation by activating both *DRP1* [127] and the OMA1 metalloprotease that mediates L-OPA1 cleavage [128]. Alternatively, L-OPA1 cleavage by YME1L is thought to be modulated by mitochondrial OXPHOS and energetic state [28], while fatty acid overload is known to trigger the ubiquitin-dependent degradation of *MFN2* [129]. These data are consistent with the changes to *MFN2*, L-OPA1 and *DRP1* protein expression we report with prolonged PPA exposure, which may yield mechanistic insight into the impairments to mitochondrial integrity we observed under the same conditions.

Importantly, mitochondrial mechanisms are not only dynamic but also specific to different cell types and developmental stages. Different phases of neuronal development may be associated with divergent responses to PPA, which limits the translatability of findings in undifferentiated cells to more mature neuronal populations. Indeed, immortalized neuroblastoma cells and mature neurons may differ substantially with respect to their bioenergetic state and susceptibility to metabolic and oxidative stress. Thus, future work in differentiated SH-SY5Y cells, as well as more biologically accurate models such as human-derived neuronal stem cells, primary neurons or 3D organoid systems, would improve the translatability of these findings in a clinical context. Nevertheless, reductionist preclinical models can yield valuable mechanistic insights to complement or inform clinical research into complex neurological conditions. Elucidating the mitochondrial remodeling associated with PPA in vitro contributes to a broader effort to understand the mitochondrial component of neurological disease that could be harnessed for the development of novel therapeutic strategies.

## 5. Conclusions

This study contributes novel insights into the molecular mechanisms that remodel mitostasis under PPA stress in the SH-SY5Y system. We report time- and dose-dependent effects on cell viability and metabolism, driven by a remodeling of mitochondrial dynamics and bioenergetics. Our data highlight a complex, multifactorial molecular network that governs mitochondrial adaptation and dysfunction under stress. PPA broadly downregulates transcripts associated with mitochondrial biogenesis, fission and fusion. More subtle changes to protein expression suggest a decreased capacity for fusion, driven by L-OPA1 and MFN2, with changes to mitochondrial fission and connectivity mediated by DRP1 and LC3-II. On a functional level, PPA induces compensatory mitochondrial network remodeling by upregulating fission and fusion after 24 h. However, prolonged exposure significantly perturbs mitochondrial network integrity and bioenergetics, marked by impairments to mitochondrial respiration, glycolysis and fatty acid metabolism. Collectively, this work emphasizes the need to better understand the relationship between gene transcription, translation, localization and function in mitochondrial phenotypes. Further characterizing the molecular mechanisms that facilitate mitochondrial remodeling in the PPA model could yield important insights into the pathophysiology of PA and ASD. In particular, understanding mitochondrial remodeling in neuronal progenitor cells may reveal novel findings pertaining to the etiological overlap between inherited metabolic diseases and complex neurological disorders.

## Data Availability

The original contributions presented in this study are included in the article, Supplementary Materials. The raw data files are available from the corresponding author upon reasonable request.

## Supplementary Materials

The following supporting information can be downloaded at https://www.mdpi.com/article/10.3390/biology15161424/s1. Supplementary File S1: Supplementary Figures and Tables [130,131,132,133,134,135,136,137]. Table S1. Details of RT-qPCR assays used to measure mtDNA copy number and mRNA expression. F = forward primer; R = reverse primer. Table S2. Details of antibodies used to measure protein expression via Western blot. Figure S1. Image pre-processing and analysis pipeline for mitochondrial network analysis. Representative slices of showing (A) Preprocessing and (B) Analysis pipelines for Mitotracker (MITO) and LC3β channels. Stacks were processed by subtracting background (rolling ball = 10 pixels), followed by 10 rounds of deconvolution using the Iterative Deconvolve plugin and a pre-processing protocol employing despeckling, smoothing and enhancing with an unsharp mask. Binarised MitoTracker masks were used to generate 2D outlines and 2D Skeletons using the MitoMorphology and Analyse Skeleton plugins respectively. Binarised LC3β Masks were filtered to remove particles smaller than 0.06 uM and volume was measured using the 3D objects counter plugin. Figure S2. Propionic acid-induced cytotoxicity in SH-SY5Y cells. Undifferentiated SH-SY5Y cells were exposed to propionic acid (PPA) in EMEM:F12 for 4, 24 and 72 h followed by MTT assays to establish windows of cytotoxicity. (A) MTT assays revealed a time- and dose-dependent decrease in cell viability in response to 3–10 mM PPA or 2 h of 100μM FCCP as a positive control (*n* = 7) where data represent mean and standard deviation. All experiments were conducted in triplicate and cell viability was calculated as the average blank-corrected absorbance of all three triplicates normalised to the average of untreated controls. Data were tested for normality using the Shapiro-Wilks test before significance was determined using one-way ANOVAs with Dunnet’s Post-Hoc Comparisons where * *p* < 0.05, ** *p* < 0.01, *** *p* < 0.001, and **** *p* < 0.0001. (B) Morphological changes in undifferentiated SH-SY5Y cells exposed to PPA for 24, 48 and 72 h were visualised using a light microscope at 20X magnification, revealing decreased cell density and elongated cell bodies with prolonged PPA exposure. Figure S3. Propionic acid disrupts transcriptional regulators of mitostasis. Reverse Transcription Quantitative Polymerase Chain Reaction (RT-qPCR) was used to measure the mRNA expression of genes involved in (A) mitochondrial metabolism and biogenesis or (B) mitochondrial fission and fusion in undifferentiated SH-SY5Y cells exposed to propionic acid (PPA). Data represent the fold-change in expression relative to untreated controls according to the ΔΔCT method (2^−ΔΔCT^). Each assay was performed in triplicate, data were tested for normality using the Shapiro-Wilks test and significance was established using one-way ANOVAs with Dunnet’s multiple comparisons or Kruskal-Wallis test with Dunn’s Post-Hoc Comparisons where * *p* < 0.05, ** *p* < 0.01, *** *p* < 0.001, and **** *p* < 0.0001. *PCCB*—Propionyl CoA Carboxylase B; *c-MYC*—*c-MYC* protooncogene; *DRP1*—Dynamin-Related Protein 1; *PGC1α*—Peroxisome Proliferator-Activated Receptor Gamma Coactivator-1 Alpha; *NRF1*—Nuclear Respiratory Factor 1; *TFAM*—Mitochondrial DNA Transcription Factor A; *MFN1*/2—Mitofusin 1/2; *OPA1*—Optic Atrophy 1; *STOML2*—Stomatin-Like Protein 2. Figure S4. *OPA1* protein expression under propionic acid stress. SH-SY5Y cells were treated with 3, 5 or 10 mM PPA for 24 or 72 h before protein extraction and quantification using western blots, where protein abundance was normalized to total protein. Fold change in protein expression following PPA treatment was calculated relative to the control (0 mM) for (A) Total OPA1 and (B) S-OPA1. Outliers were removed using the ROUT method and data tested for normality using the Shapiro-Wilks test. Significance was tested using one-way ANOVA with Dunnet’s for multiple comparisons or Kruskal-Wallis using Dunn’s for multiple comparisons; * *p* < 0.05, ** *p* < 0.01. Bars represent mean ± SD or median ± interquartile range. Data shown represent a minimum of *n* = 3 biological repeats. Original gels and blots are presented in Supplementary File S2. Figure S5. Equilibrium between fission and fusion events. SH-SY5Y cells treated with 3, 5 or 10 mM propionic acid (PPA) for 24 h were stained with TMRE and Hoechst 33342 and imaged across nine consecutive time points. (A) The average number of fission and fusion events per frame. (B) The total number of fission and fusion events over nine consecutive frames. Data represent mean ± SD for a minimum of *n* =13 cells per treatment group, * *p* < 0.05; ** *p* < 0.01. Figure S6. Maintenance of mitochondrial DNA copy number. RT-qPCR was used to measure Relative ND1 Copy Number after (A) 24 (*n* = 3) and (B) 72 h (*n* = 4) in cells treated with 3,5 and 10 mM PPA. Data represent the fold-change in expression relative to untreated controls according to the ΔΔCT method (2^−ΔΔCT^). Each assay was performed in triplicate and significance was established using one-way ANOVAs with Dunnet’s multiple comparisons or Kruskal-Wallis test with Dunn’s Post-Hoc Comparisons where * *p* < 0.05, ** *p* < 0.01, *** *p* < 0.001, and **** *p* < 0.0001. Figure S7. Impaired ATP production and altered metabolic phenotype. The ATP Rate Assay was used to quantify total, mitochondrial and glycolytic ATP production in SH-SY5Y cells after 72 h at 5 mM PPA. Data represent oxygen consumption rate (OCR) (A–C), extracellular acidification rate (ECAR) (D–F), and metabolic phenotype (G,H) measured over the duration of the assay. Here, baseline refers to basal metabolic state while stressed refers to metabolic state after the addition of Oligomycin and Rot/AA. Data represent triplicates from one biological repeat where significant differences were measured with two-way ANOVAs and Tukey’s Post Hoc Comparisons; * *p* < 0.05, ** *p* < 0.01, *** *p* < 0.001, and **** *p* < 0.0001. Table S3. MitoPlate S1 metabolites showing significant substrate utilization. Substrate utilization was assessed by calculating the change in absorbance 560 nm values between 30 min and 4 h for controls and cells exposed to propionic acid (PPA) for 72 h. Data represent the metabolite, treatment condition, corresponding *p*-value derived from unpaired two-tailed *t*-tests and q-value following FDR correction for multiple comparison for the metabolites showing significant utilization in either control (*n* = 7) or PPA treated (*n* = 4) samples. Table S4. Maximum metabolic rate for TCA substrates of interest. The maximum instantaneous rate for TCA metabolites of interest, measured as ∆Absorbance 560 nm/hr. Data were assessed for normality using the Shapiro-Wilks test and significance was measured using Welches or Mann-Whitnney *t*-tests with Benjamini Hochberg correction. Table S5. Ordinary Least Square Regression Analysis for TCA substrates of interest. Linear models were defined as y = β0 + β1 (time in hrs) + β2 (condition) + β3 (time × condition) where y = ∆Absorbance at 560 nm over Time (hrs) for SH-SY5Y cells in the absence (control) or presence of 5 mM Propionic Acid for 72 h (PPA). Residuals were assessed for normality using the Shapiro-Wilks test and heteroscedasticity was assessed using the Breusch-Pagan Lagrange Multiplier test. Supplementary File S2: Original uncropped Western blot images corresponding to Figure 2 and Figure S4. Total protein blots (left) and corresponding ECL blots (right) for the proteins are shown. Red boxes indicate the bands used for densitometric quantification. PPA concentrations of samples are indicated above the corresponding lanes (mM PPA). Duration of PPA exposure is indicated for each set of blots. For each protein, the protein ladder bands (10–180 kDa) are indicated for the first total protein blot and the specific protein bands are indicated in the first ECL blot. Unlabeled lanes are not relevant to the specific protein being quantified. A. OPA1, B. MFN2, C. DRP1, D. LC3-II and E. TOM20. Supplementary File S3: Descriptive Summary and Statistics.

## Abbreviations

The following abbreviations are used in this manuscript:

ΔΨm	mitochondrial membrane potential
AA	amino acid
ASD	Autism Spectrum Disorder
*c-MYC*	*c-MYC* protooncogene
ECAR	extracellular acidification rate
ETC	electron transport chain
ETF/QOR	electron transfer flavoprotein quinone oxidoreductase
FA	fatty acid
FOA	fatty acid oxidation
FCCP	carbonyl cyanide 4-(trifluoromethoxy) phenylhydrazone
IMD	inherited metabolic disease
LC3-II	Microtubule-Associated Protein 1 Light Chain 3 Beta
MICOS	Mitochondrial Contact Site and Cristae Organizing System
*MFN1/2*	Mitofusin 1/2
mtDNA	mitochondrial DNA
*NRF1*	Nuclear Respiratory Factor 1
OCR	oxygen consumption rate
*OPA1*	Optic Atrophy 1
OXPHOS	oxidative phosphorylation
PA	Propionic Acidemia
PARK	parkin
*PCCB*	Propionyl CoA Carboxylase B
*PGC1α*	Peroxisome Proliferator-Activated Receptor Gamma Coactivator-1 Alpha
*PINK1*	PTEN-induced putative kinase 1
PPA	Propionic Acid
TCA	Tricarboxylic Acid
*TFAM*	Mitochondrial DNA Transcription Factor A
*TOMM20*	Translocase Of Outer Mitochondrial Membrane 20
*STOML2*	Stomatin-Like Protein 2
