# Supplementary material for: Propionic Acid Remodels Mitochondrial Metabolism in SH-SY5Y Cells
Source: Biology (Basel). 2026 Aug 18;15(16):1424. doi: 10.3390/biology15161424 (PMC13509392; doi:10.3390/biology15161424)
Supplement: Supplementary file 1 [file biology-15-01424-s001.zip › biology-4472852-supplementary/Supplementary File S1.pdf]

Supplementary File 1

Table S1. Details of RT-qPCR assays used to measure mtDNA copy number and mRNA expression. F= forward primer; R = reverse primer.

Gene	Primer Sequence	Reference	Master Mix	Cycling Conditions
B2M (DNA)	F: 5′-CCAGCAGAGAATGGAAAGTCAA-3′	Grady *et al.*, 2014 [130]	1µL DNA 0.5µL Primers	1 x (95°C 5min) 40 x (95°C 15s; 60°C 30s)
	R: 5′-TCTCTCTCCATTCTTCAGTAAGTCAACT-3′			
ND1	F: 5′-CCCTAAAACCCGCCACATCT-3′			
	R: 5′-GAGCGATGGTGAGAGCTAAGGT-3′			
B2M (RNA)	F: 5′-CCAGCAGAGAATGGAAAGTCAA-3′	Grady *et al.*, 2014 [130]	0.6µL cDNA, 0.75µL Primers	Dependent on GOI
	R: 5′-TCTCTCTCCATTCTTCAGTAAGTCAACT-3′			
CMYC	F: 5'- ACCACCAGCAGCGACTCTGA-3′	Wang *et al.*, 2018 [66]	1µL cDNA 1µL Primers	1 x (95°C 60s) 40X (95°C 10s; 60°C 30s; 72°C 10s)
	R: 5'- TCCAGCAGAAGGTGATCCAGACT-3′			
NRF1	F: 5′-TTGAGTCTAATCCATCTATCCG-3′	Pinho *et al.*, 2022 [131]	0.8µL cDNA 0.5µL Primers	
	R: 5′-TACTTACGCACCACATTCTC-3′			
TFAM	F: 5′-GTCACTGCCTCATCCACC-3′	Ye *et al.*, 2022 [132]		
	R: 5′-CCGCCCTATAAGCATCTT-3′			
18S	F: 5′-CCTGGATACCGCAGCTAGGA-3′	Kang *et al.*, 2018 [133]	1µL cDNA 1µL Primers	1 x (95°C, 5min) 35 x (95°C 30s; 60°C 45s; 72°C 50s)
	R: 5′-GCGGCGCAATACGAATGCCCC-3′			
PGC1α	F: 5′-CCGAGAATTCATGGAGCAAT -3'	Kim *et al.*, 2019 [22]	0.5µL cDNA 0.5µL Primers	
	R: 5′- TTTCTGTGGGTTTGGTGTGA-3'			
DRP1	F: 5′-TCAACCTCCGCGTCTACTC-3′	Kosmider *et al.*, 2019 [134]	0.8µL cDNA 0.5µL Primers	1 x (95°C 60s) 35 x (95°C 15s; 63°C 30s; 72°C 10s)
	R: 5′-GATCTGGAACTCGATGTCGGG-3′			
MFN1	F: 5′-GAGGTGCTATCTCGGAGACAC-3′			
	R: 5′-GCCAATCCCACTAGGGAGAAC-3′			
MFN2	F: 5′-TGGCTCAAGACTATAAGCTGCG-3′	Duraisamy *et al.*, 2019 [135]	0.8µL cDNA, 0.4 µL Primers	
	R: 5′-GAGGACTACTGGAGAAGGGTGG-3′			
STOML2	F: 5′-GTGACTCTCGACAATGTAAC-3′	Hu *et al.*, 2018 [136]	0.8 µL cDNA, 0.5 µL Primers	1 x (95°C 10min) 40 x (95°C 15s; 62°C 30s)
	R: 5′-TGATCTCATAACGGAGGCAG-3′			

OPA1	F: 5'- AGCCTCGCAGGAATTTTTGG-3'	Bean *et al.*, 2021 [137]	0.6 µL cDNA, 0.75 µL Primers	1 x (95°C 60s) 40 x (95°C 10s; 60°C 40s; 72°C 20s)
	R: 5′-AGCCGATCCTAGTATGAGATAGC-3'			
PCCB	F: 5′- AGGCAGTCTGTCAGGAGCACAT-3′	Origene #HP200504	1 µL cDNA 0.5 µL Primers	
	R: 5′- CCAGAGTCATTCAGCCCAATCAC-3′			
	R: 5′-AGCCGATCCTAGTATGAGATAGC-3'			

Table S2: Details of antibodies used to measure protein expression via western blot.

Antibody	Source	Working Concentration
	Primary Antibodies	
**OPA1 (D7C1A) Rabbit mAb**	67589, Cell Signalling Technology	1:1000
**Mitofusin-2 (D1E9) Rabbit mAb**	11925, Cell Signalling Technology	1:1000
**DRP1 (D6C7) Rabbit mAb**	8570, Cell Signalling Technology	1:1000
**TOMM20 (M01, clone 4F3) Mouse mAb**	H00009804-M01, Abnova	1:2000
**LC3II**	83506, Cell Signalling Technology	1:1000
	Secondary Antibodies	
**Goat anti-rabbit IgG,HRP-linked Antibody**	7074, Cell Signalling Technology	1:10 000
**Rabbit anti-mouse, IgG HRP-linked antibody**	PA1-28568, Thermofisher Scientific	1:2 000

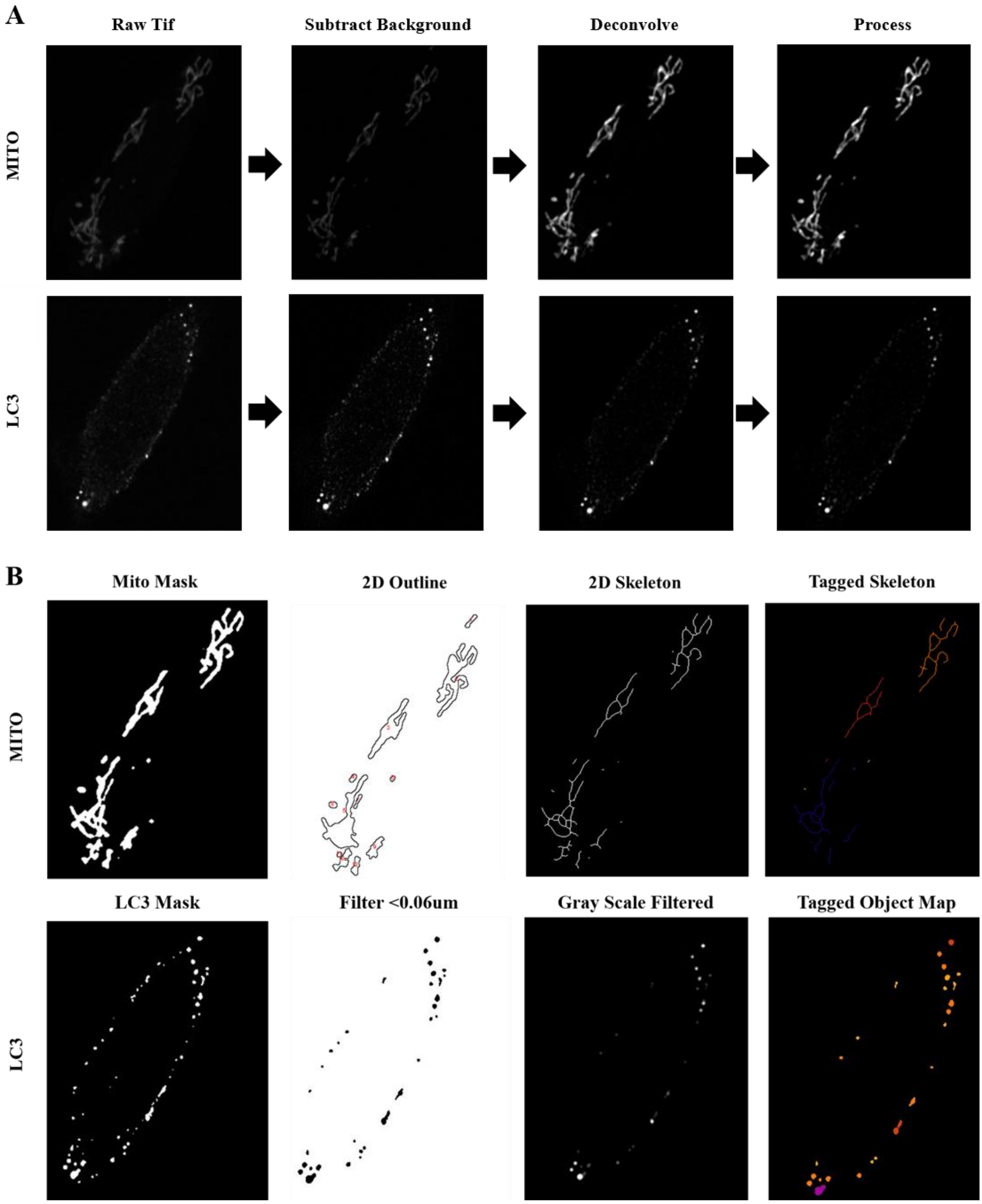

Figure S1. Image Pre-processing and Analysis Pipeline for Mitochondrial Network Analysis. Representative slices of showing A) Preprocessing and B) Analysis pipelines for Mitotracker (MITO) and LC3β channels. Stacks were processed by subtracting background (rolling ball = 10 pixels), followed by 10 rounds of deconvolution using the Iterative Deconvolve plugin and a pre-processing protocol employing despeckling, smoothing and enhancing with an unsharp mask. Binarised MitoTracker masks were used to generate 2D outlines and 2D Skeletons using the MitoMorphology and Analyse Skeleton plugins respectively. Binarised LC3β Masks were filtered to remove particles smaller than 0.06uM and volume was measured using the 3D objects counter plugin.

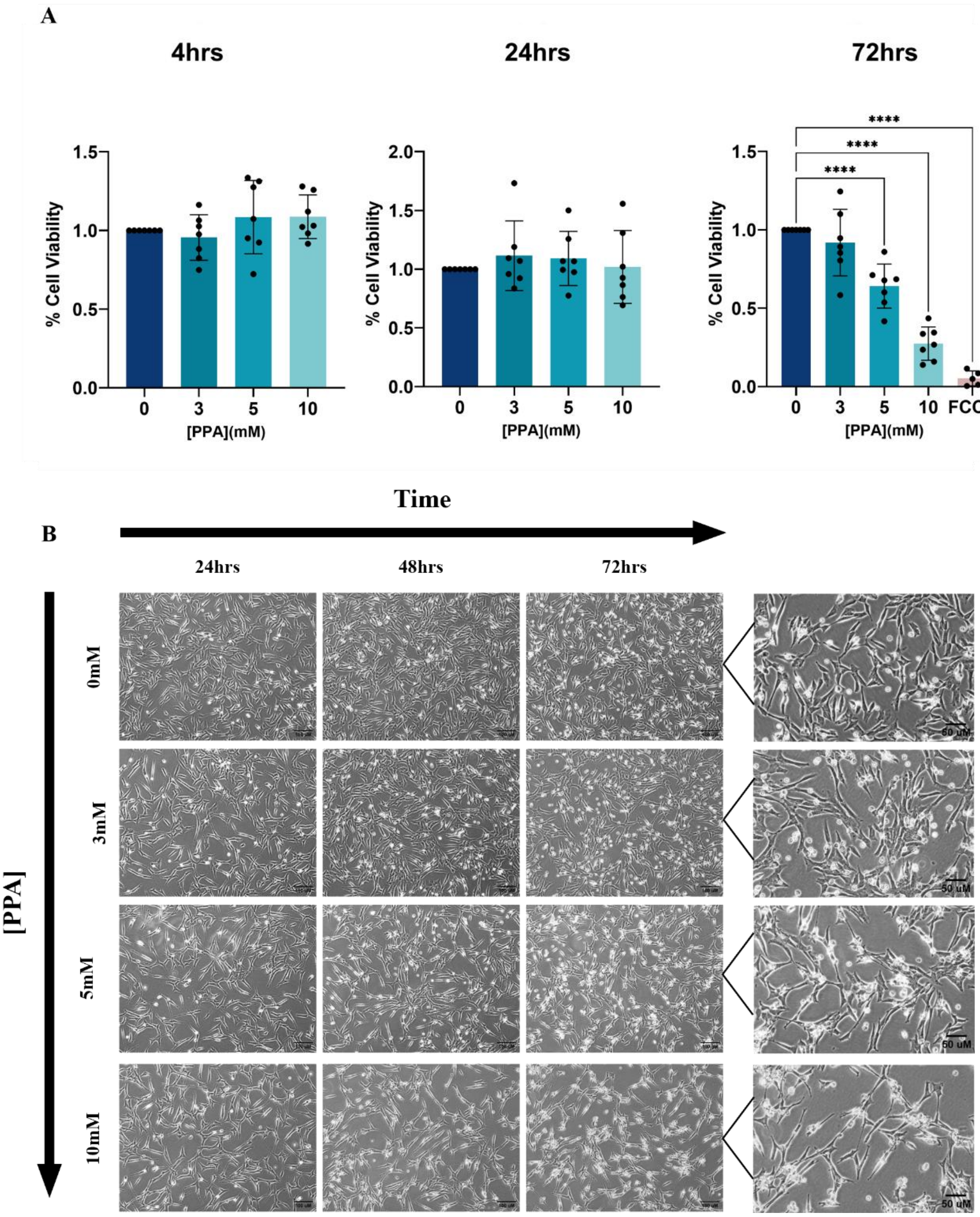

Figure S2. Propionic Acid-Induced Cytotoxicity in SH-SY5Y Cells. Undifferentiated SH-SY5Y cells were exposed to propionic acid (PPA) in EMEM:F12 for 4, 24 and 72 hours followed by MTT assays to establish windows of cytotoxicity. **A)** MTT assays revealed a time- and dose-dependent decrease in cell viability in response to 3-10mM PPA or 2 hours of 100µM FCCP as a positive control (n=7) where data represent mean and standard deviation. All experiments were conducted in triplicate and cell viability was calculated as the average blank-corrected absorbance of all three triplicates normalised to the average of untreated controls. Data were tested for normality using the Shapiro-Wilks test before significance was determined using one-way ANOVAs with Dunnet's Post-Hoc Comparisons where $*p<0.05$, $**p<0.01$, $***p<0.001$, and $****p<0.0001$. **B)** Morphological changes in undifferentiated SH-SY5Y cells exposed to PPA for 24, 48 and 72 hours were visualised using a light microscope at 20X magnification, revealing decreased cell density and elongated cell bodies with prolonged PPA exposure.

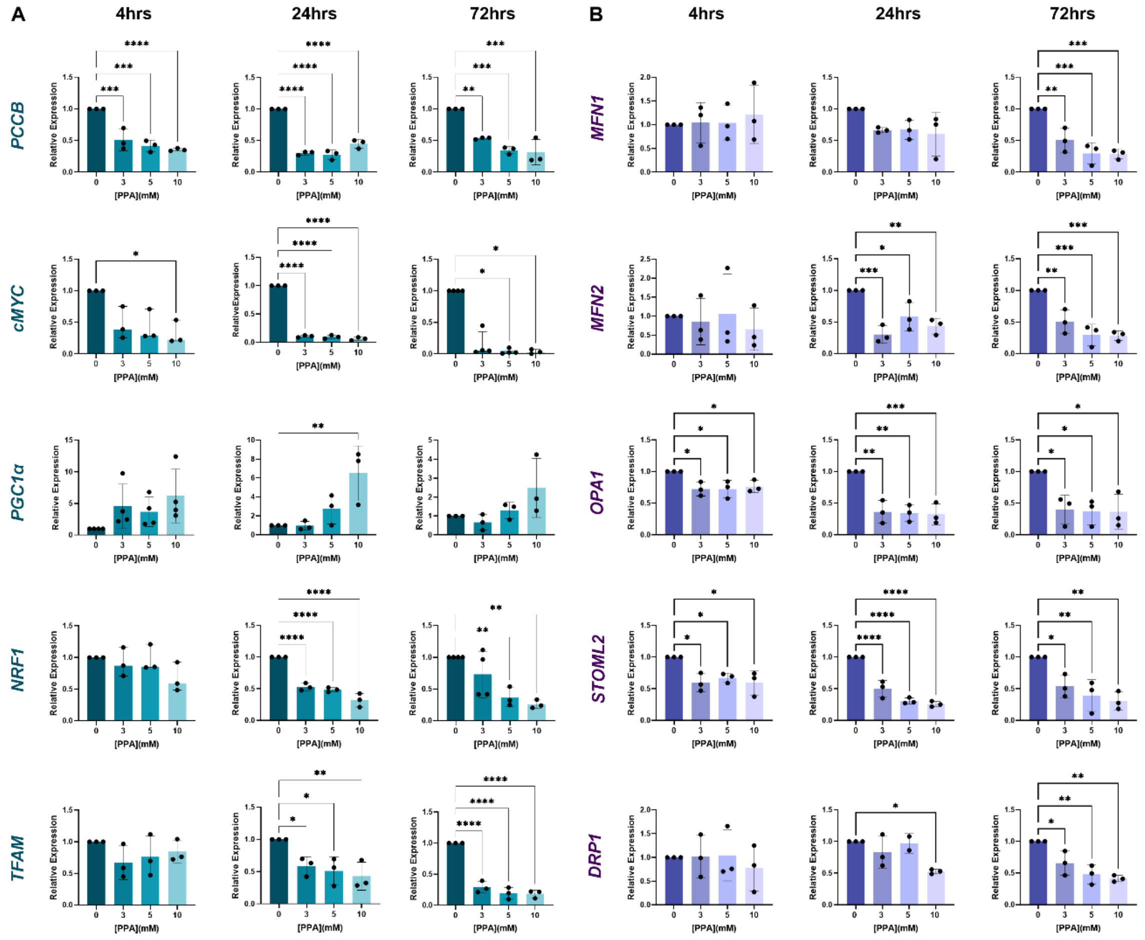

Figure S3. Propionic Acid Disrupts Transcriptional Regulators of Mitostasis. Reverse Transcription Quantitative Polymerase Chain Reaction (RT-qPCR) was used to measure the mRNA expression of genes involved in **A)** mitochondrial metabolism and biogenesis or **B)** mitochondrial fission and fusion in undifferentiated SH-SY5Y cells exposed to propionic acid (PPA). Data represent the fold-change in expression relative to untreated controls according to the ΔΔCT method ($2^{-\Delta\Delta CT}$). Each assay was performed in triplicate, data were tested for normality using the Shapiro-Wilks test and significance was established using one-way ANOVAs with Dunnet's multiple comparisons or Kruskal-Wallis test with Dunn's Post-Hoc Comparisons where *$p<0.05$, **$p<0.01$, ***$p<0.001$, and ****$p<0.0001$. PCCB - Propionyl CoA Carboxylase B; c-MYC - c-MYC protooncogene; PGC1α - Peroxisome Proliferator-Activated Receptor Gamma Coactivator-1 Alpha; NRF1 - Nuclear Respiratory Factor 1; TFAM - Mitochondrial DNA Transcription Factor A; MFN1/2 – Mitofusin 1/2; OPA1 - Optic Atrophy 1; STOML2 - Stomatin-Like Protein 2; DRP1 - Dynamin-Related Protein 1.

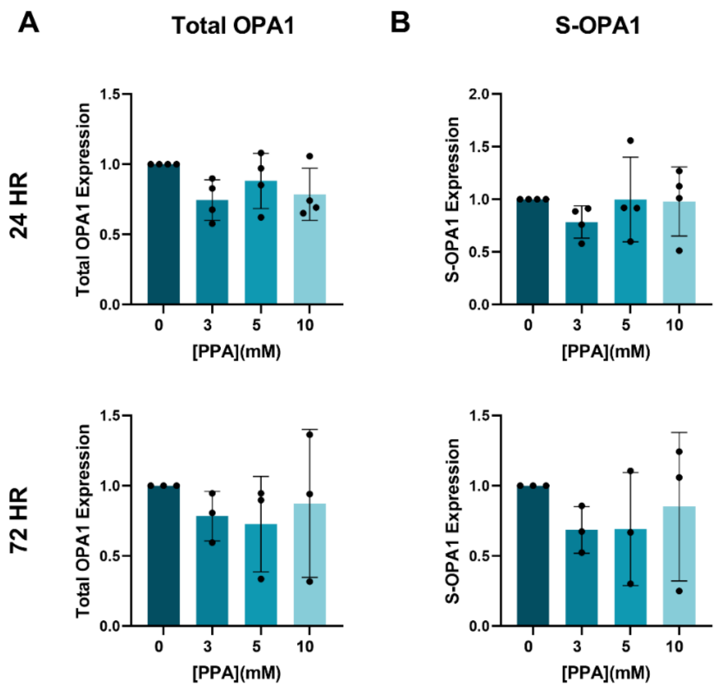

Figure S4. OPA1 Protein Expression Under Propionic Acid Stress. SH-SY5Y cells were treated with 3, 5 or 10mM PPA for 24 or 72 hours before protein extraction and quantification using western blots, where protein abundance was normalized to total protein. Fold change in protein expression following PPA treatment was calculated relative to the control (0mM) for A) Total OPA1 and B) S-OPA1. Outliers were removed using the ROUT method and data tested for normality using the Shapiro-Wilks test. Significance was tested using one-way ANOVA with Dunnet's for multiple comparisons or Kruskal-Wallis using Dunn's for multiple comparisons; *p<0.05, **p<0.01. Bars represent mean ± SD or median ± interquartile range. Data shown represent a minimum of n = 3 biological repeats. Original gels and blots are presented in Appendix A.

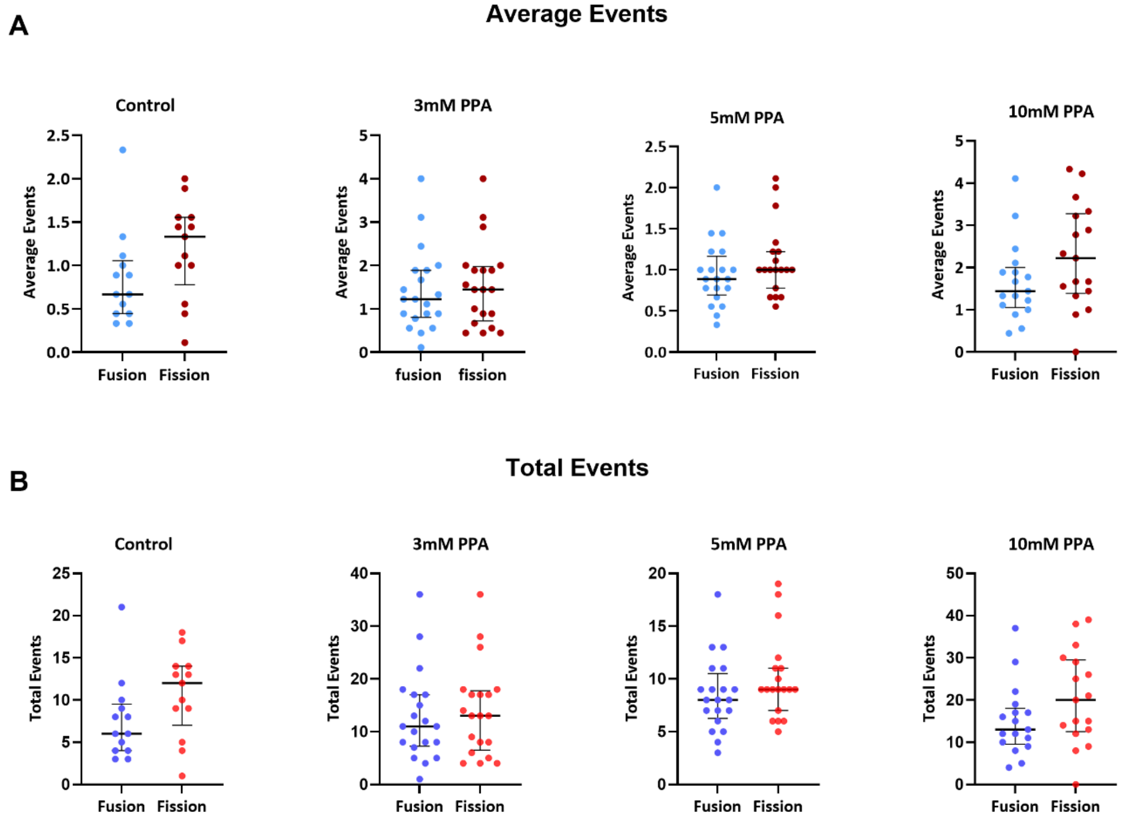

Figure S5. Equilibrium Between Fission and Fusion Events. SH-SY5Y cells treated with 3, 5 or 10mM propionic acid (PPA) for 24 hours were stained with TMRE and Hoechst 33342 and imaged across nine consecutive time points. A) The average number of fission and fusion events per frame. B) The total number of fission and fusion events over nine consecutive frames. Data represent mean ± SD for a minimum of n =13 cells per treatment group, *p<0.05; **p<0.01.

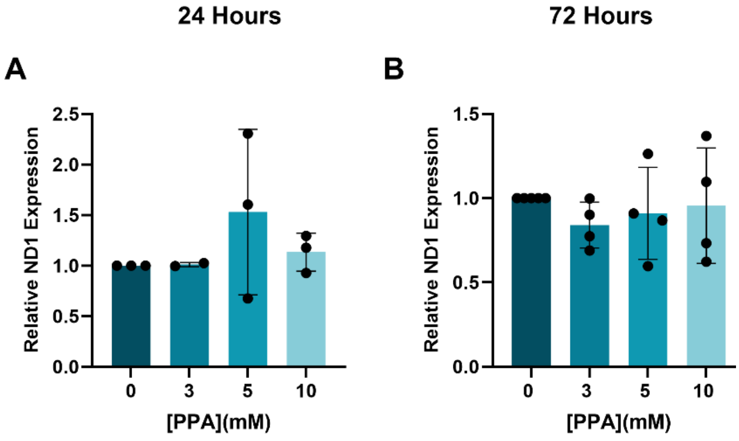

Figure S6. Maintenance of Mitochondrial DNA Copy Number. RT-qPCR was used to measure Relative ND1 Copy Number after **A)** 24 (n=3) and **B)** 72 hours (n=4) in cells treated with 3,5 and 10mM PPA. Data represent the fold-change in expression relative to untreated controls according to the ΔΔCT method ($2^{-\Delta\Delta CT}$). Each assay was performed in triplicate and significance was established using one-way ANOVAs with Dunnet's multiple comparisons or Kruskal-Wallis test with Dunn's Post-Hoc Comparisons where *p<0.05, **p<0.01, ***p<0.001, and ****p<0.0001.

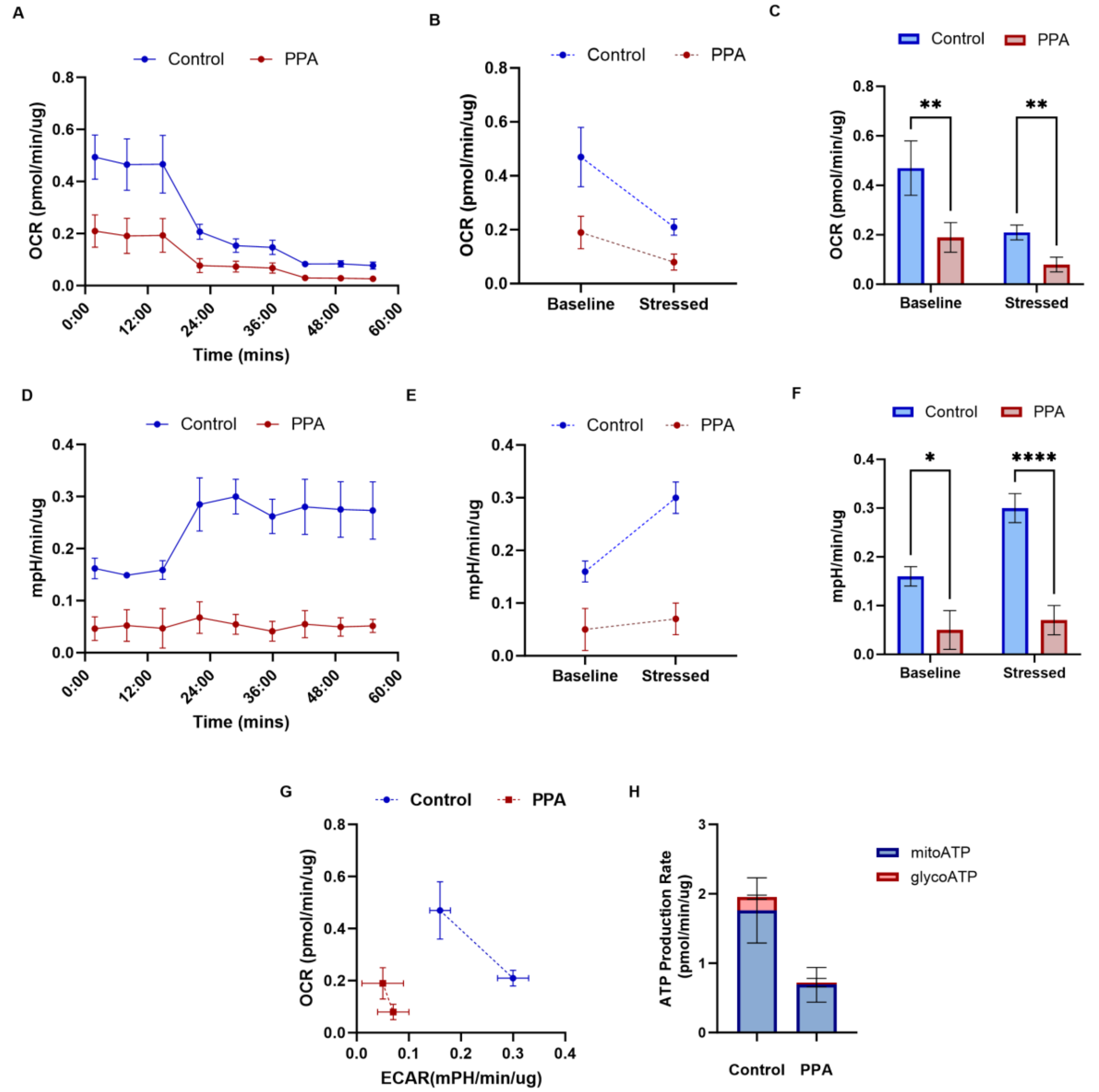

Figure S7. Impaired ATP Production and Altered Metabolic Phenotype. The ATP Rate Assay was used to quantify total, mitochondrial and glycolytic ATP production in SH-SY5Y cells after 72 hours at 5mM PPA. Data represent oxygen consumption rate (OCR) **(A-C)**, extracellular acidification rate (ECAR) **(D-F)**, and metabolic phenotype **(G, H)** measured over the duration of the assay. Here, baseline refers to basal metabolic state while stressed refers to metabolic state after the addition of Oligomycin and Rot/AA. Data represent triplicates from one biological repeat where significant differences were measured with two-way ANOVAs and Tukey's Post Hoc Comparisons; *p<0.05, **p<0.01, ***p<0.001, and ****p<0.0001.

Table S3. MitoPlate S1 Metabolites Showing Significant Substrate Utilization. Substrate utilization was assessed by calculating the change in absorbance 560nm values between 30 minutes and 4 hours for controls and cells exposed to propionic acid (PPA) for 72 hours. Data represent the metabolite, treatment condition, corresponding p-value derived from unpaired two-tailed t-tests and q-value following FDR correction for multiple comparison for the metabolites showing significant utilization in either control (n = 7) or PPA treated (n = 4) samples.

Metabolite	Control			PPA		
	t.stat	p-value	FDR	t.stat	p-value	FDR
cis-Aconitic Acid	-4.12	5.22E-04	**2.70E-03**	-2.31	6.78E-02	1.91E-01
D,L-α-Glycerol-PO4	-3.50	3.25E-03	**1.12E-02**	-2.29	5.32E-02	1.83E-01

Fumaric Acid	-7.58	2.23E-06	**1.38E-05**	-4.71	2.63E-03	**2.04E-02**
Glycogen	-3.59	1.70E-03	**6.69E-03**	-2.45	4.83E-02	1.83E-01
L-Malic Acid	-8.14	1.89E-07	**1.95E-06**	-5.40	1.36E-03	**1.41E-02**
Palmitoyl-D,L-Carnitine+L-Malic	-3.03	7.14E-03	**2.21E-02**	-3.02	1.51E-02	6.67E-02
Pyruvic Acid+L-Malic Acid 100uM	-9.10	1.74E-07	**1.95E-06**	-5.56	9.18E-04	**1.41E-02**
Pyruvic Acid	-3.62	1.73E-03	**6.69E-03**	-4.17	4.79E-03	**2.48E-02**
Succinic Acid	-15.10	1.58E-11	**4.89E-10**	-7.42	2.53E-04	**7.84E-03**
α-Keto-Glutaric Acid	-7.75	9.56E-07	**7.41E-06**	-4.73	4.79E-03	**2.48E-02**

Table S4. Maximum metabolic rate for TCA Substrates of Interest. The maximum instantaneous rate for TCA metabolites of interest, measured as ΔAbsorbance 560nm/hr. Data were assessed for normality using the Shapiro-Wilks test and significance was measured using Welches or Mann-Whitnney t-tests with Benjamini Hochberg correction.

**Metabolite**	**Max Rate (Δ560nm/hr)**						
	**Normality**	**Mean/Median**		**SD/IQR**		**P value**	**FDR**
		**Control**	**PPA**	**Control**	**PPA**		
Aconitic Acid	Y	0.002	0.003	0.001	0.001	0.286	0.327
Fumaric Acid	N	0.003	0.006	0.002	0.004	0.109	0.218
L-Malic Acid	Y	0.005	0.007	0.001	0.002	0.173	0.276
Palmitoyl-D,L-Carnitine+L-Malic	N	0.001	0.002	0.000	0.002	**0.010**	**0.038**
Pyruvic Acid+L-Malic Acid 100uM	Y	0.003	0.003	0.001	0.001	0.281	0.327
Pyruvic Acid	N	0.001	0.003	0.001	0.002	**0.006**	**0.038**
Succinic Acid	Y	0.007	0.008	0.002	0.002	0.403	0.403
α-Keto-Glutaric Acid	Y	0.004	0.006	0.001	0.002	0.098	0.218

Table S5. Ordinary Least Square Regression Analysis for TCA Substrates of Interest. Linear models were defined as $y=\beta 0 + \beta 1(\text{time in hrs}) + \beta 2(\text{condition}) + \beta 3(\text{time} \times \text{condition})$ where y = ΔAbsorbance at 560nm over Time (hrs) for SH-SY5Y cells in the absence (control) or presence of 5mM Propionic Acid for 72 hours (PPA). Residuals were assessed for normality using the Shapiro-Wilks test and heteroscedasticity was assessed using the Breusch-Pagan Lagrange Multiplier test.

**Metabolite**	**Control**		**PPA**		**P Intercept**	**P Interaction**	**FDR Interaction**
	**Intercept**	**Slope**	**Intercept**	**Slope**			
cis-Aconitic Acid	0.025	0.018	0.005	0.054	0.105	**0.004**	**0.016**
Fumaric Acid	0.007	0.104	-0.039	0.132	0.091	0.250	0.286
α-Keto-Glutaric Acid	0.039	0.094	-0.025	0.135	**0.001**	**0.018**	**0.037**
L-Malic Acid	0.006	0.149	-0.035	0.168	0.087	0.397	0.397
Palmitoyl-D,L-Carnitine	-0.037	0.012	-0.026	0.041	0.418	**0.006**	**0.017**
Pyruvic Acid	0.023	0.020	0.005	0.074	0.209	**0.000**	**0.000**
Pyruvic Acid + L-Malic Acid	-0.011	0.069	0.013	0.047	0.085	0.066	0.106
Succinic Acid	0.027	0.143	-0.043	0.174	**0.002**	0.144	0.192
