# Supplementary material for: Propionic Acid Remodels Mitochondrial Metabolism in SH-SY5Y Cells
Source: Biology (Basel). 2026 Aug 18;15(16):1424. doi: 10.3390/biology15161424 (PMC13509392; doi:10.3390/biology15161424)
Supplement: Supplementary file 1 [file biology-15-01424-s001.zip › biology-4472852-supplementary/Supplementary File S2.pdf]

Supplementary File 2

Original uncropped Western blot images corresponding to Figure 2. and S4. Total protein blots (left) and corresponding ECL blots (right) for the proteins are shown. Red boxes indicate the bands used for densitometric quantification. PPA concentrations of samples are indicated above the corrosponding lanes (mM PPA). Duration of PPA exposure is indicated for each set of blots. For each protein, the protein ladder bands (10-180 kDa) are indicated for the first total protein blot and the specific protein bands are indicated in the first ECL blot. Unlabelled lanes are not relevant to the specific protein being quantified. **A.** OPA1, **B.** MFN2, **C.** DRP1, **D.** LC3-II and **E.** TOM20.

A. OPA1

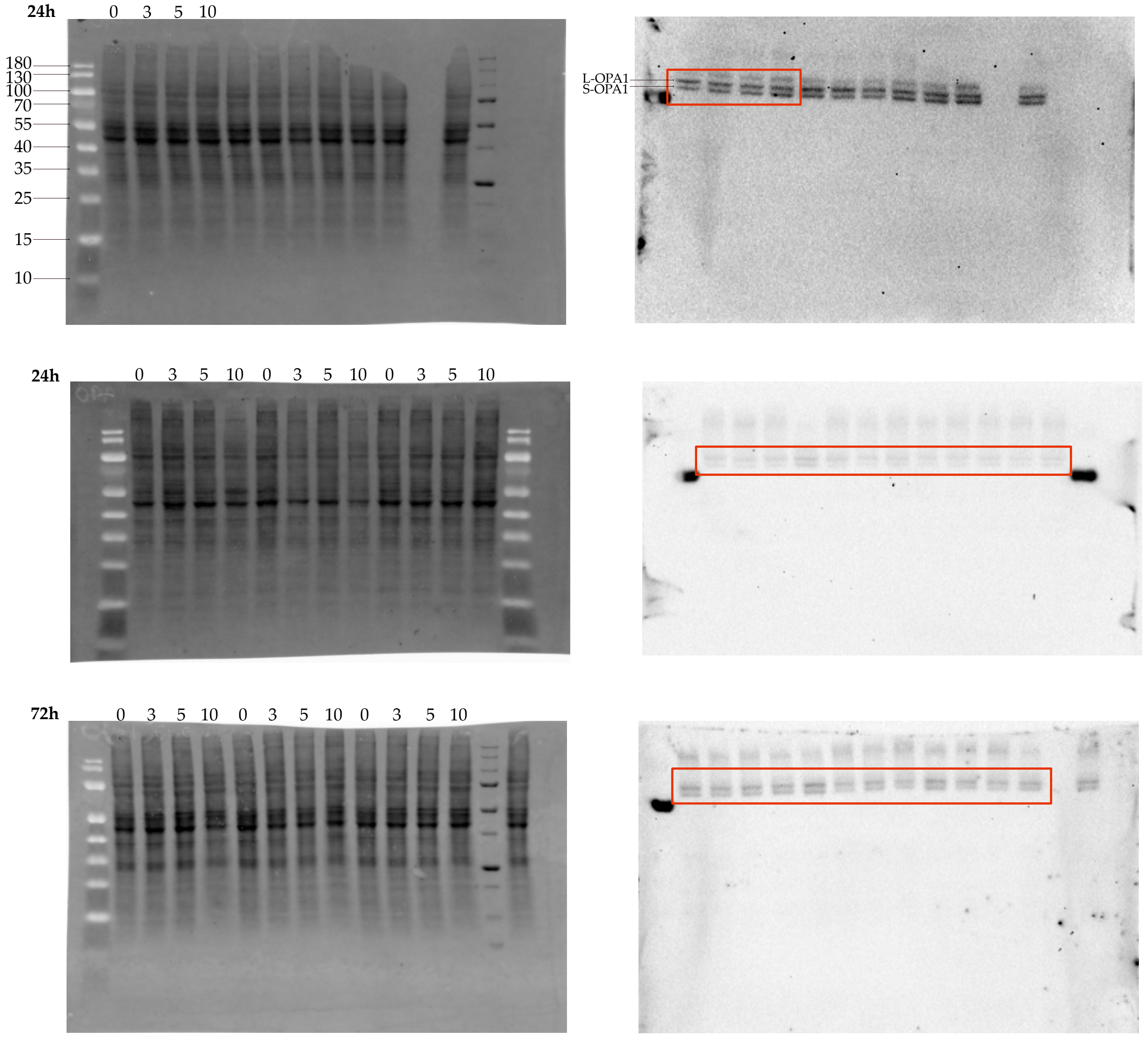

B. MFN2

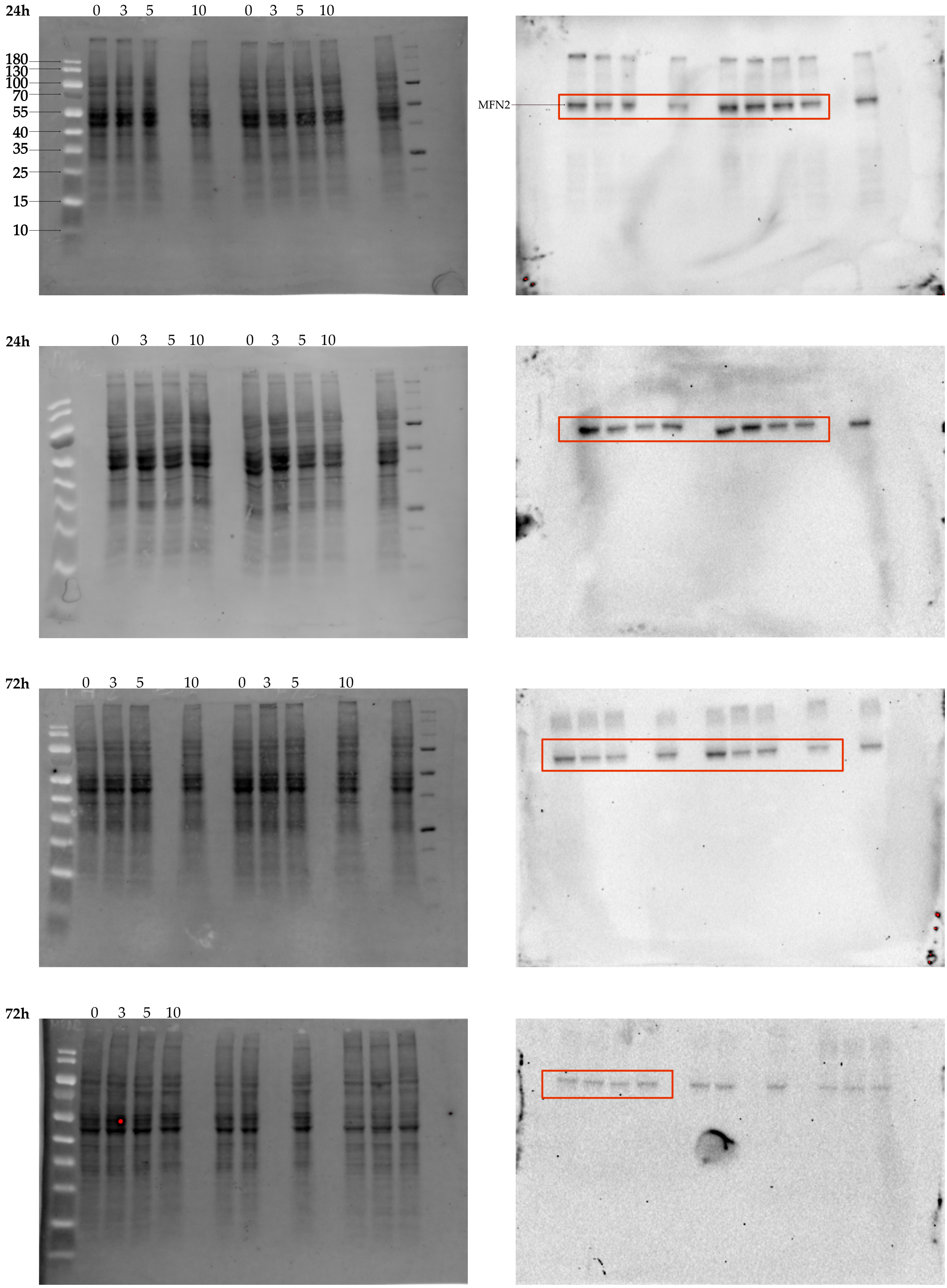
24h
0 3 5 10 0 3 5 10
180
130
100
70
55
40
35
25
15
10
MFN2
24h
0 3 5 10 0 3 5 10
72h
0 3 5 10 0 3 5 10
72h
0 3 5 10

C. DRP1

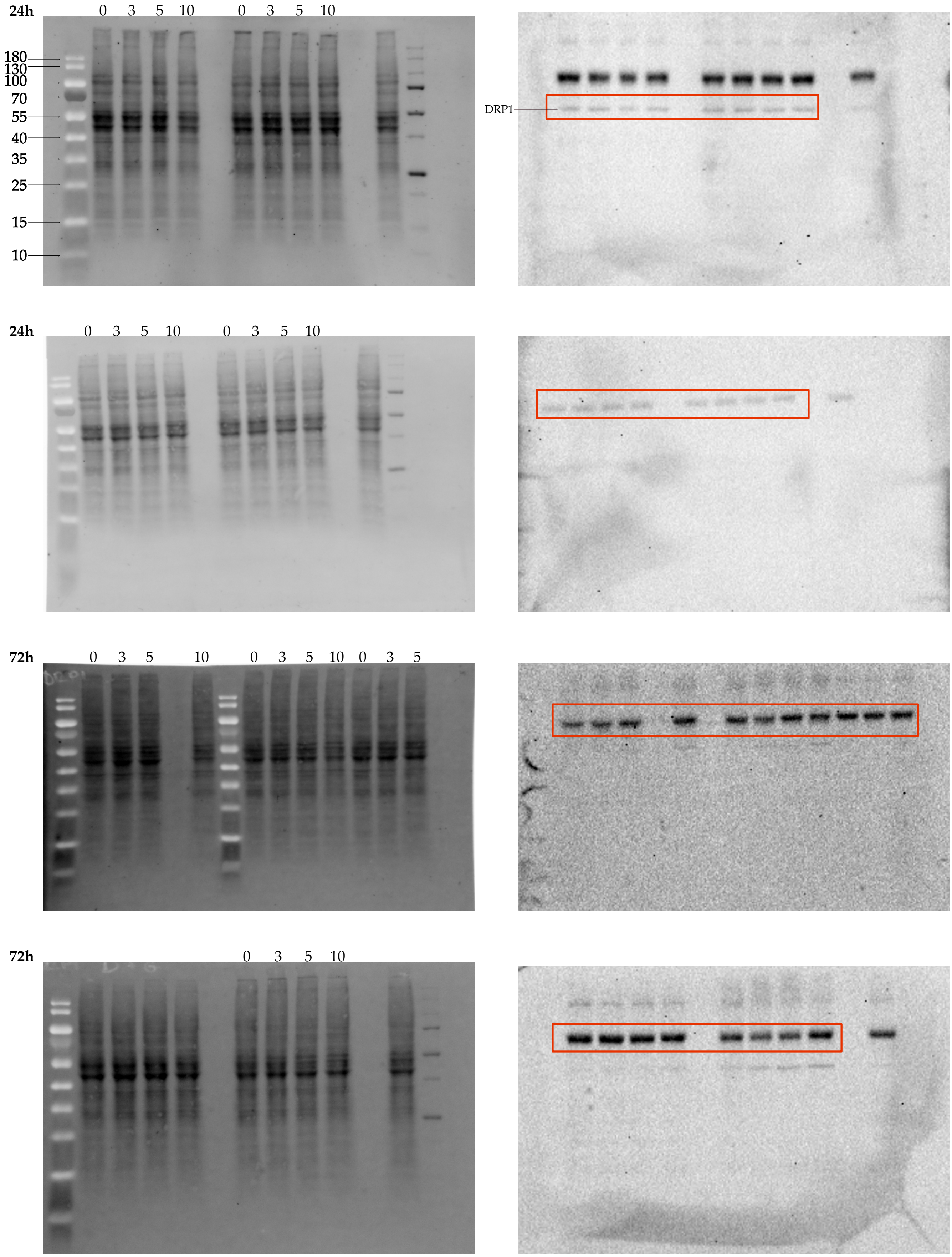
24h
0 3 5 10 0 3 5 10
180
130
100
70
55
40
35
25
15
10
DRP1
24h
0 3 5 10 0 3 5 10
72h
0 3 5 10 0 3 5 10 0 3 5
72h
0 3 5 10

D. LC3-II

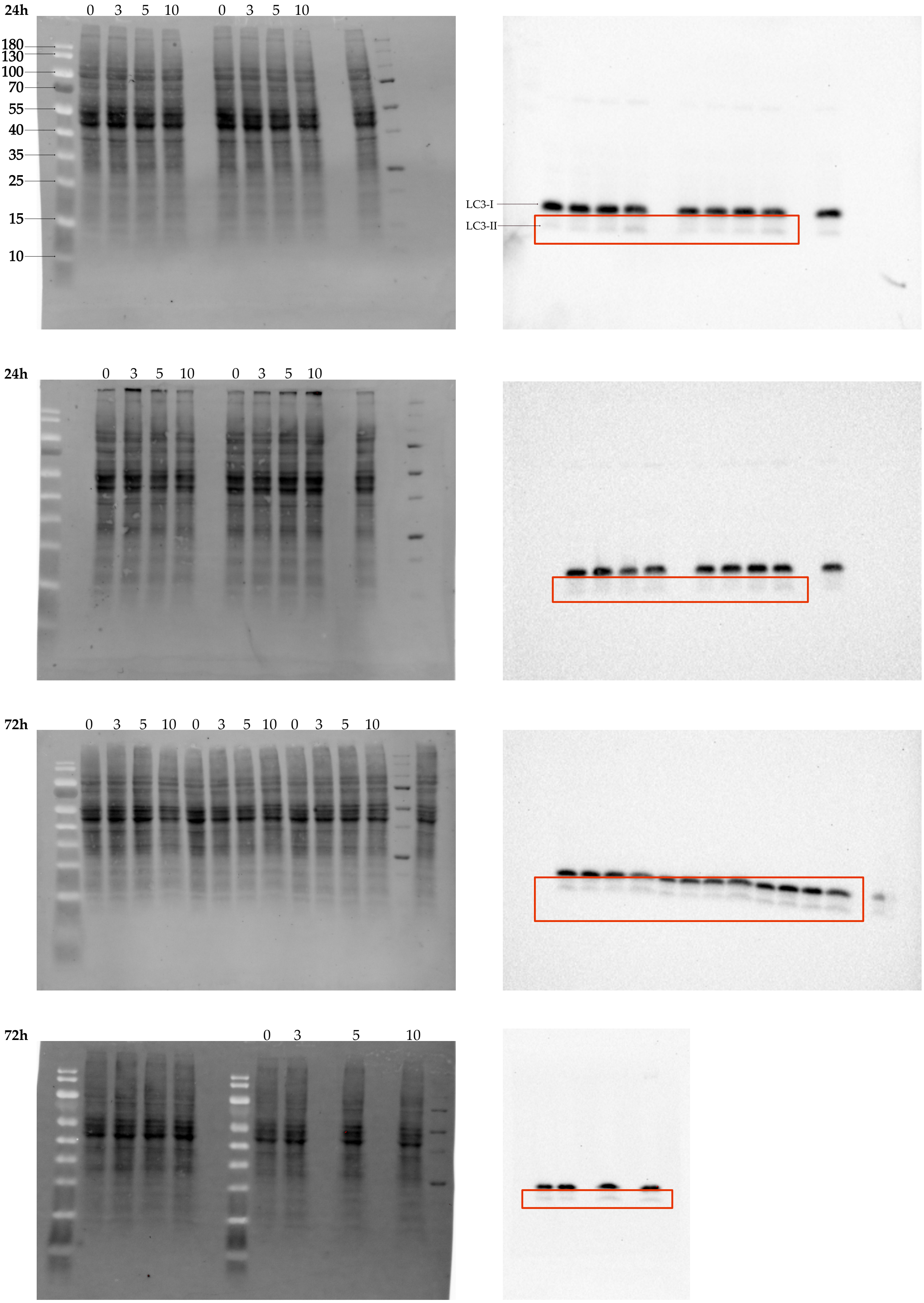
24h
0 3 5 10 0 3 5 10
180
130
100
70
55
40
35
25
15
10
LC3-I
LC3-II
24h
0 3 5 10 0 3 5 10
72h
0 3 5 10 0 3 5 10 0 3 5 10
72h
0 3 5 10

E. TOM20

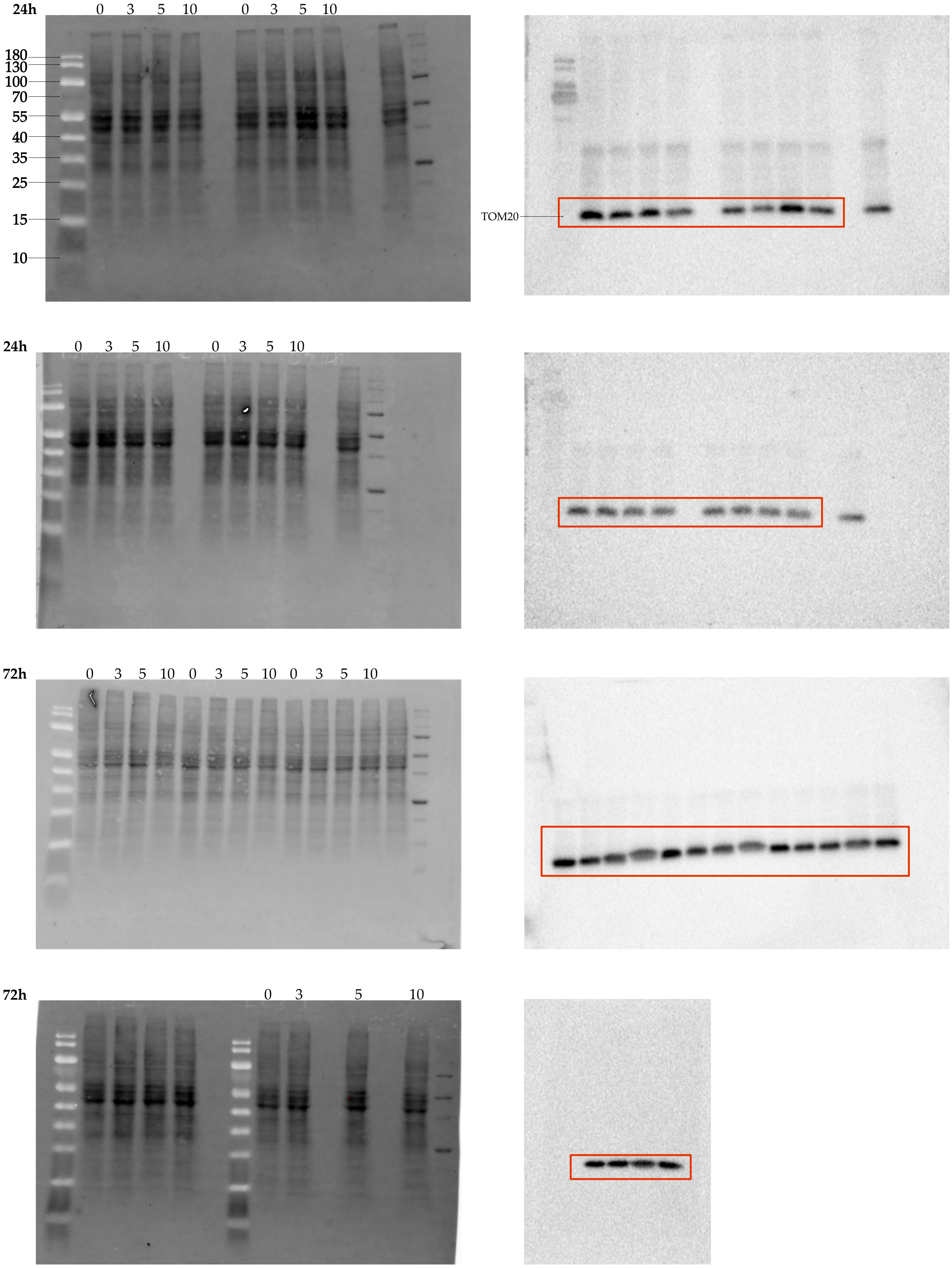
24h
0 3 5 10 0 3 5 10
180
130
100
70
55
40
35
25
15
10
TOM20
24h
0 3 5 10 0 3 5 10
72h
0 3 5 10 0 3 5 10 0 3 5 10
72h
0 3 5 10
